# Effects of Ablation Versus Drug Therapy on Quality of Life by Sex in Atrial Fibrillation: Results From the CABANA Trial

**DOI:** 10.1161/JAHA.122.027871

**Published:** 2023-01-23

**Authors:** Emily P. Zeitler, Yanhong Li, Adam P. Silverstein, Andrea M. Russo, Jeanne E. Poole, Melanie R. Daniels, Hussein R. Al‐Khalidi, Kerry L. Lee, Tristram D. Bahnson, Kevin J. Anstrom, Douglas L. Packer, Daniel B. Mark, for the CABANA Investigators

**Affiliations:** ^1^ Dartmouth Health and The Dartmouth Institute Lebanon NH USA; ^2^ Duke Clinical Research Institute, Duke University Durham NC USA; ^3^ Division of Cardiovascular Disease Cooper Medical School of Rowan University Camden NJ USA; ^4^ University of Washington Medical Center Seattle WA USA; ^5^ Department of Biostatistics and Bioinformatics Duke University Durham NC USA; ^6^ Division of Cardiology, Department of Medicine Duke University Medical Center Durham NC USA; ^7^ University of North Carolina Chapel Hill NC USA; ^8^ Mayo Clinic Rochester MN USA

**Keywords:** atrial fibrillation, catheter ablation, quality of life, sex, Atrial Fibrillation, Clinical Studies, Quality and Outcomes

## Abstract

**Background:**

Women with atrial fibrillation (AF) demonstrate more AF‐related symptoms and worse quality of life (QOL). Whether increased use of ablation in women reduces sex‐related QOL differences is unknown. Sex‐related outcomes for ablation versus drug therapy was a prespecified analysis in the CABANA (Catheter Ablation Versus Antiarrhythmic Drug Therapy for Atrial Fibrillation) trial.

**Methods and Results:**

Symptoms were assessed periodically over 60 months with the Mayo AF‐Specific Symptom Inventory (MAFSI) frequency score, and QOL was assessed with the Atrial Fibrillation Effect on Quality of Life (AFEQT) summary and component scores. Women had lower baseline QOL scores than men (mean AFEQT scores 55.9 and 65.6, respectively). Ablation patients improved more than drug therapy patients with similar treatment effect by sex: AFEQT 12‐month mean adjusted treatment difference in women 6.1 points (95% CI, 3.5–8.6) and men 4.9 points (95% CI, 3.0–6.9). Participants with baseline AFEQT summary scores <70 had greater QOL improvement, with a mean treatment difference at 12 months of 7.6 points for women (95% CI, 4.3–10.9) and 6.4 points for men (95% CI, 3.3–9.4). The mean adjusted difference in MAFSI frequency score between women randomized to ablation versus drug therapy at 12 months was −2.5 (95% CI, −3.4 to −1.6); for men, the difference was −1.3 (95% CI, −2.0 to −0.6).

**Conclusions:**

Compared with drug therapy for AF, ablation resulted in more QOL improvement in both sexes, primarily driven by improvements in those with lower baseline QOL. Ablation did not eliminate the AF‐related QOL gap between women and men.

**Registration:**

URL: https://www.clinicaltrials.gov; Unique identifier: NCT00911508.

**Nonstandard Abbreviations and Acronyms**
AFEQT: Atrial Fibrillation Effect on Quality of Life
CABANA: Catheter Ablation Versus Antiarrhythmic Drug Therapy for Atrial Fibrillation
MAFSI: Mayo AF‐Specific Symptom Inventory

**Clinical Perspective**

**What Is New?**

- Women with atrial fibrillation (AF) enrolled in the CABANA (Catheter Ablation Versus Antiarrhythmic Drug Therapy for Atrial Fibrillation) trial had quality of life improvements with catheter ablation relative to drug therapy of equivalent magnitude to that seen in men.
- Women with AF had worse AF‐related quality of life than men both at baseline and at 12 months.
- Magnitude of quality of life benefit was larger for both men and women in subjects with lower (worse) baseline scores.

**What Are the Clinical Implications?**

- Despite clinically effective ablation, women with AF averaged persistently lower follow‐up quality of life scores than men with AF.

Women with atrial fibrillation (AF) generally report worse quality of life (QOL) compared with men, with more AF symptoms and more associated functional impairment.^1^, ^2^ The causes of these differences remain uncertain. Multiple clinical trials have shown that catheter ablation is significantly more effective in reducing or eliminating AF compared with drug‐based rhythm control strategies.^3^ However, observational studies have reported that women get offered ablation less often than men.^4^, ^5^, ^6^ The American College of Cardiology Committee on Cardiovascular Disease in Women recently questioned whether this apparent disparity in the use of ablation might account for at least part of the explanation for worse QOL in women with AF.^7^ The CABANA (Catheter Ablation Versus Antiarrhythmic Drug Therapy for Atrial Fibrillation) trial enrolled women in a higher proportion (37%) than most cardiovascular clinical trials, and provides an opportunity to examine the proposition that when women are offered ablation at the same rate as men, the disparities in AF QOL and symptoms are substantially attenuated.

CABANA was a National Institutes of Health–funded, randomized controlled trial designed to test the hypothesis that ablation‐based therapy of AF is more effective than drug‐based therapy for the reduction of a composite end point consisting of death, disabling stroke, serious bleeding, or cardiac arrest.^8^ The major clinical outcomes from ablation versus drug therapy for AF in CABANA were similar by sex.^9^ A preliminary report of 12‐month QOL outcomes by sex in CABANA demonstrated comparable relative benefits from ablation in men and women.^10^ The present report provides a comprehensive description of the effects of ablation versus drug therapy on QOL outcomes by sex in CABANA.

## Methods

CABANA is a National Institutes of Health/National Heart, Lung, and Blood Institute–sponsored trial, and the trial data sets will be made public via the National Institutes of Health BioLINCC website.

### CABANA Trial Overview

The recruitment, treatment, and outcome assessment strategies in CABANA have been reported previously.^8^, ^11^ Briefly, 2204 patients with new‐onset or undertreated symptomatic AF were enrolled between 2009 and 2016 at 126 international clinical sites. To qualify for enrollment, patients were required to be either ≥65 years old or <65 years old, with 1 or more risk factors for stroke. Patients were randomized to a treatment strategy of catheter ablation versus drug therapy, and the primary outcome was a composite of death, disabling stroke, serious bleeding, or cardiac arrest. Median follow‐up was 48.5 months. All enrolled patients provided written informed consent, and the study protocol was approved at each site by the local institutional review board or ethics committee.

### Primary Clinical Results by Sex

We have previously reported a comparison of major clinical outcomes by sex and treatment group in CABANA.^9^ CABANA randomized 819 women (413 ablation, 406 drug therapy) and 1385 men (695 ablation, 690 drug therapy). Baseline characteristics by sex are shown in Table S1. Women were older than men (median ages 69 years versus 67 years, respectively). Women were also more symptomatic at baseline; 48% were in Canadian Cardiovascular Society class 3 or 4 versus 39% of men, and 42% were in New York Heart Association class II or III compared with 32% of men. Women had a higher baseline prevalence of paroxysmal AF (50% versus 39% for men). Treatment complications were quite infrequent for both sexes. For the CABANA primary composite clinical outcome (death, disabling stroke, serious bleeding, or cardiac arrest), the ablation–drug therapy hazard ratio (HR) was 1.01 for women (95% CI, 0.62–1.65) and 0.73 for men (95% CI, 0.51–1.05) (interaction *P*=0.299). For all‐cause mortality in women who had an ablation, the drug therapy HR was 0.62 (95% CI, 0.33–1.16). For men, the HR was 0.92 (95% CI, 0.60–1.41) (interaction *P*=0.313). Freedom from AF recurrence was better with ablation for both sexes; for women the HR was 0.64 (95% CI, 0.51–0.82), and for men the HR was 0.48 (95% CI, 0.40–0.58) (interaction *P*=0.06).

### QOL Assessment

Assessment of long‐term QOL outcomes was a prespecified secondary objective for the CABANA research program. Two QOL measures were prespecified as coprimary QOL end points, the Atrial Fibrillation Effect on Quality of Life (AFEQT) questionnaire and the Mayo AF‐Specific Symptom Inventory (MAFSI). These assessments were performed at baseline and then 3, 12, 24, 36, 48, and 60 months after randomization. The 12‐month assessments were designated as the primary QOL comparisons. Detailed methods related to QOL assessment and the primary QOL intention‐to‐treat comparisons between randomized groups have been reported previously.^10^

### AF Effect on QOL Questionnaire

The AFEQT is a validated QOL assessment instrument specific to AF.^12^ The 21‐item AFEQT assessment produces a summary score calculated from 18 of the items as well as scored subscale results for each of 3 domains: symptoms, daily activities, and treatment concern. Each of these 4 measures is scored from 0 to 100, with 100 indicating no AF‐related disability and 0 indicating complete disability. For both the summary score and the subscale domains, a change of 5 points for an individual patient has been identified as clinically meaningful.^13^

### Mayo AF‐Specific Symptom Inventory

The modified MAFSI questionnaire used in CABANA is a 10‐item AF symptom checklist that asks about the frequency of AF‐related symptoms.^14^ The frequency score output ranges from 0 to 40, and higher scores indicate more severe AF symptoms. In prior CABANA analysis of QOL based on MAFSI, a change of 1.6 (frequency) in any individual patient was considered clinically meaningful, because this represented approximately one‐fourth of the pooled baseline standard deviation in the overall population.^15^

### Statistical Analysis

All analyses were performed by intention to treat. Baseline characteristics are reported as percentages for discrete variables or mean (SD) and/or median (25th–75th percentiles) for continuous variables.

Statistical analyses of the AFEQT and MAFSI end points follow the same methods as the primary CABANA QOL report.^10^ Briefly, the mixed model for repeated measures was used to analyze the AFEQT and MAFSI end points. Baseline and month 3, 12, 24, 36, 48, and 60 follow‐up scores were included as dependent variables; time, treatment, sex, time by treatment, time by sex, treatment by sex, and time by treatment by sex were modeled as fixed effects. Parameters were estimated using restricted maximum likelihood with an unstructured covariance matrix and Kenward‐Roger degrees of freedom approximation. No missing value imputations were performed on the outcome variables, because the mixed model for repeated measures does not require complete follow‐up outcomes data for each patient.

Point estimates for each treatment group as well as treatment group mean differences (ablation, drug therapy) with 95% CI were generated using the ESTIMATE Statement with the SAS PROC MIXED procedure. No adjustments were made in the analyses for multiple comparisons. Instead, we limited the number of formal statistical tests reported and focused primarily on the estimated treatment effect sizes and associated precision. *P* values, where provided, are intended solely as adjunctive interpretive aids with statistical significance understood to be a heuristic, reflecting the consistency of the data with the null hypothesis.^16^

Prior QOL analysis in CABANA used post hoc baseline AFEQT scores subgroups <70, 70 to 89, and ≥90 to identify severely symptomatic, mildly to moderately symptomatic, and asymptomatic patients with AF, respectively, to examine whether treatment response magnitude at 12 months was affected by baseline scores.^10^ We chose the same cut points in the present analysis to examine if response magnitude subdivided by baseline QOL scores varied by sex.

AFEQT and MAFSI outcomes are reported by treatment group and sex both overall and according to the AFEQT subgroups expressed as mean difference (ablation minus drug therapy) with 95% CI. Results are displayed as bar graphs and histograms (unadjusted) as well as forest plots (adjusted mean difference).

All analyses were performed using SAS software version 9.4 or later (SAS Institute).

## Results

### Baseline QOL

No clinically relevant differences in baseline AFEQT or MAFSI by treatment group were seen within sex subgroups (Tables 1 and 2). However, men enrolled in CABANA had more favorable unadjusted baseline QOL scores, with average scores about 10 points higher than for women (AFEQT summary scores 65.6 and 55.9, respectively). Approximately three‐quarters of the women randomized in CABANA had severe AF symptoms at the time of enrollment, with a baseline AFEQT summary score <70 (Table S2). By comparison, among men, only 51% had a baseline AFEQT summary score <70 (Table S3).

**Table 1:** QOL Outcomes by Treatment Assignment in CABANA for Women

QOL measure	Catheter ablation, N=413		Drug therapy, N=406		
	No.	Mean (SD)	No.	Mean (SD)	Mean adjusted difference, CA minus drug (95% CI)
AFEQT summary score					
Baseline	408	56.7 (19.7)	400	55.1 (19.0)	1.7 (−1.0 to 4.4)
3 mo	358	75.3 (19.7)	360	70.7 (21.4)	4.5 (1.7 to 7.2)
12 mo	338	81.5 (18.1)	334	75.3 (20.3)	6.1 (3.5 to 8.6)
24 mo	302	82.3 (17.3)	284	76.9 (20.7)	5.4 (2.7 to 8.0)
36 mo	225	81.4 (18.2)	207	79.6 (18.8)	2.8 (0.0 to 5.6)
48 mo	168	81.7 (17.5)	159	79.1 (18.7)	3.4 (0.2 to 6.5)
60 mo	123	80.6 (18.3)	114	78.6 (20.3)	2.7 (−1.1 to 6.4)
All follow‐up	1514	80.1 (18.5)	1458	75.7 (20.5)	4.1 (2.0 to 6.2)
MAFSI frequency score					
Baseline	401	13.5 (6.0)	395	13.9 (6.2)	−0.4 (−1.2 to 0.5)
3 mo	336	9.0 (6.4)	333	10.9 (6.6)	−2.1 (−3.0 to −1.2)
12 mo	312	8.1 (6.4)	315	10.5 (6.4)	−2.5 (−3.4 to −1.6)
24 mo	279	8.0 (6.2)	267	10.1 (6.8)	−2.0 (−3.0 to −1.1)
36 mo	205	8.6 (6.5)	205	8.9 (6.5)	−0.7 (−1.7 to 0.4)
48 mo	153	8.8 (6.5)	141	9.0 (6.4)	−0.5 (−1.7 to 0.7)
60 mo	105	8.1 (6.7)	110	9.5 (7.3)	−1.4 (−2.8 to −0.1)
All follow‐up	1390	8.4 (6.4)	1371	10.1 (6.6)	−1.5 (−2.3 to −0.8)

AFEQT indicates Atrial Fibrillation Effect on Quality of Life; CA, catheter ablation; CABANA, Catheter Ablation Versus Antiarrhythmic Drug Therapy; MAFSI, Mayo AF‐Specific Symptom Inventory; and QOL, quality of life.

**Table 2:** QOL Outcomes by Treatment Assignment in CABANA for Men

QOL measure	Catheter ablation, N=695		Drug therapy, N=690		
	No.	Mean (SD)	No.	Mean (SD)	Mean adjusted difference, CA minus drug (95% CI)
AFEQT summary score					
Baseline	676	66.5 (20.1)	678	67.8 (20.0)	−1.3 (−3.4 to 0.8)
3 mo	613	82.4 (17.4)	623	79.9 (19.1)	2.2 (0.1 to 4.3)
12 mo	577	89.3 (14.8)	569	84.1 (16.4)	4.9 (3.0 to 6.9)
24 mo	554	89.0 (15.0)	514	84.5 (17.3)	3.8 (1.8 to 5.8)
36 mo	420	88.7 (13.7)	398	86.2 (16.0)	2.2 (0.2 to 4.3)
48 mo	308	88.7 (14.4)	314	85.7 (16.7)	2.8 (0.4 to 5.1)
60 mo	206	89.5 (13.9)	206	85.8 (17.1)	2.6 (−0.2 to 5.4)
All follow‐up	2678	87.5 (15.4)	2624	83.8 (17.4)	3.1 (1.5 to 4.7)
MAFSI frequency score					
Baseline	668	10.8 (6.1)	666	10.8 (6.3)	−0.0 (−0.7 to 0.6)
3 mo	561	6.3 (5.4)	561	7.6 (6.2)	−1.3 (−2.0 to −0.6)
12 mo	516	5.3 (5.5)	516	6.6 (5.7)	−1.3 (−2.0 to −0.6)
24 mo	480	5.1 (5.2)	457	6.7 (6.0)	−1.5 (−2.2 to −0.8)
36 mo	366	5.2 (5.1)	354	6.7 (6.3)	−1.6 (−2.4 to −0.8)
48 mo	271	5.2 (5.5)	278	6.1 (5.9)	−1.0 (−1.9 to −0.2)
60 mo	174	4.4 (4.5)	185	5.5 (5.0)	−1.3 (−2.3 to −0.2)
All follow‐up	2368	5.4 (5.3)	2351	6.7 (6.0)	−1.3 (−1.9 to −0.8)

AFEQT indicates Atrial Fibrillation Effect on Quality of Life; CA, catheter ablation; CABANA, Catheter Ablation Versus Antiarrhythmic Drug Therapy; MAFSI, Mayo AF‐Specific Symptom Inventory; and QOL, quality of life.

When examined by the 4 most commonly reported MAFSI symptom components (palpitations/ heart fluttering/racing, shortness of breath, unable to exercise, and tired/lack of energy), no differences between treatment groups were seen at baseline (Table S4). Tired/lack of energy was the symptom reported by the greatest number of women in both treatment groups (92%), followed by palpitations/heart fluttering/racing (88%), shortness of breath (83%), and inability to exercise (73%). In comparison, the rates of these symptoms in men were tired/lack of energy (86%), palpitations/heart fluttering/racing (75%), shortness of breath (75%), and inability to exercise (58%).

### QOL Outcomes

#### AF Effect on QOL Questionnaire

**Figure 1:**
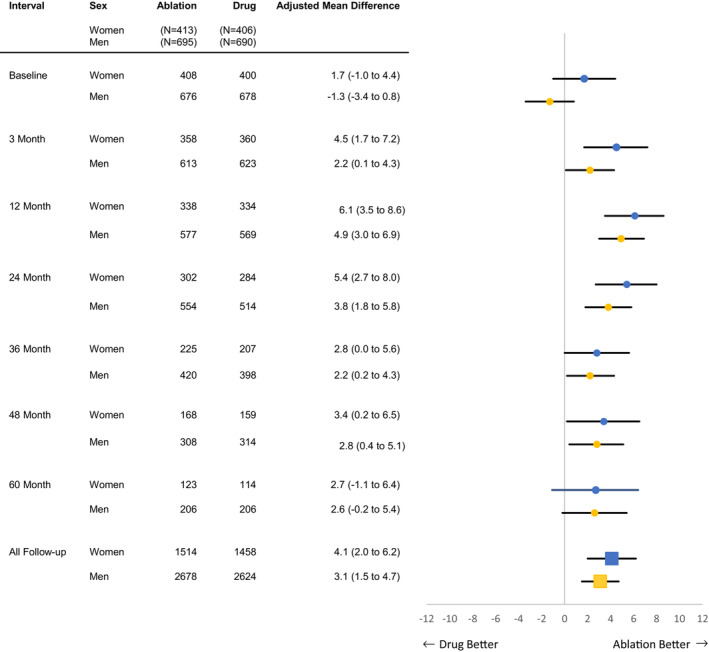
Differences in AFEQT questionnaire summary score between treatment groups. Blue is women and yellow is men. AFEQT indicates Atrial Fibrillation Effect on Quality of Life.

For women, at 12 months after randomization, the mean (±SD) AFEQT summary score randomized to ablation was 81.5±18.1 compared with 75.3±20.3 in the drug therapy arm, corresponding to a mean adjusted difference of 6.1 (95% CI, 3.5–8.6; *P*<0.001) (Table 1). The corresponding results in men were 89.3±14.8 in the ablation arm and 84.1±16.4 in the drug therapy arm, with a mean adjusted 12‐month difference of 4.9 (95% CI, 3.0–6.9; *P*<0.001) (Table 2). For women, the treatment difference narrowed after 24 months, and at 5 years the treatment group scores were ablation 80.6±18.3 and drug therapy 78.6±20.3 (mean difference 2.7 [95% CI, −1.1 to 6.4]) (Figure 1). The same pattern was seen in men, with 5‐year AFEQT summary scores of 89.5±13.9 for ablation, 85.8±17.1 for drug therapy, with a mean difference of 2.6 (95% CI, −0.2 to 5.4) (Figure 1).

As was seen in the overall CABANA cohort, the larger mean QOL improvements from ablation over drug therapy reported at 12 months in the group with baseline AFEQT summary score <70 narrowed over the final 3 years of follow‐up. This reduction in benefit from ablation appears to be attributable mostly to improvements in mean AFEQT summary score in the drug therapy arm (Table S5).

**Figure 2:**
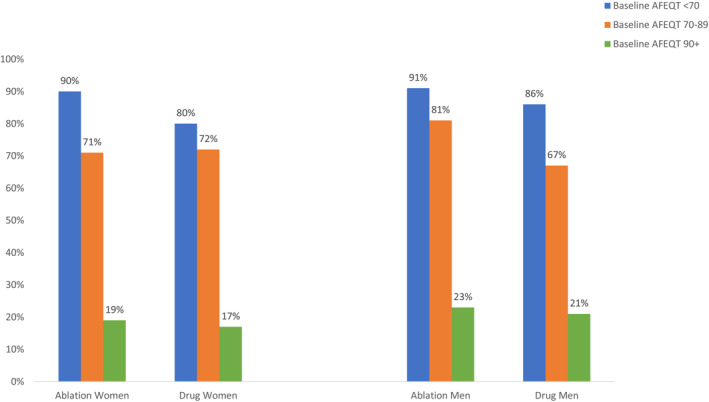
Proportion of patients achieving 5 points or more improvement in AFEQT questionnaire summary score at 12 months by baseline summary score, treatment strategy, and sex. AFEQT indicates Atrial Fibrillation Effect on Quality of Life.

When examined at the patient level, a 5‐point or more improvement in the AFEQT summary score at 12 months was more likely in the ablation group compared with the drug therapy group for women, but only among patients whose baseline score was <70 (Figure 2). For men, the largest incremental ablation advantage in 12‐month AFEQT scores was seen in patients with baseline scores 70 to 89 (Figure 2).

When examined by AFEQT subdomains of symptoms, daily activities, and treatment concern, the pattern was similar for both sexes. Early QOL advantages accrued by the ablation arm narrowed in later follow‐up (Table S6, Figures S1 through S3).

When stratified by baseline AFEQT summary score into groups with better (≥70) and worse (<70) baseline scores, larger QOL benefit with ablation was seen for both sexes in the group with worse baseline AFEQT summary score. For women, the mean adjusted difference in this subgroup at 12 months with ablation was 7.6 (95% CI, 4.3–10.9). For men, the mean adjusted difference was 6.4 (95% CI, 3.3–9.4) (Table S5, Figure S4). Adjusted mean AFEQT score treatment differences out to 60 months were comparable for men and women (Figure S4). In the group with better baseline AFEQT summary score (≥70), the average AFEQT summary score among men was improved in the ablation group (mean adjusted 12‐month difference 3.7 [95% CI, 1.8–5.5]; overall difference 1.4 [95% CI, 0.1–2.9]). No difference was seen in the subgroup of women with a score of ≥70 (mean adjusted 12‐month difference −0.7 [95% CI, −4.0 to 2.6]; the overall follow‐up assessments difference was 0.4 [95% CI, −2.4 to 3.2]) (Figure S5).

#### Mayo AF‐Specific Symptom Inventory

The mean adjusted difference in MAFSI frequency score at 12 months between women randomized to ablation versus drug therapy was −2.5 (95% CI, −3.4 to −1.6) (Table 1), whereas for men, the difference was −1.3 (95% CI, −2.0 to −0.6) (Table 2).

Of the 4 most common AF symptoms reported in MAFSI by women, the magnitude of symptom improvement as reflected in the percent of patients achieving never or rare symptom frequency at 12 months with ablation relative to drug therapy was largest for tired/lack of energy, inability to exercise, and palpitations/heart fluttering/racing but not shortness of breath (Table S4). Men showed an opposite pattern, with shortness of breath showing the largest percentage of patients achieving never or rare symptom frequency at 12 months with ablation over drug therapy (Table S4).

## Discussion

Four findings in the present report are particularly noteworthy. First, the mean baseline QOL scores for women in CABANA were worse than those for the men, and the women had a greater symptom burden compared with men. The mean baseline AFEQT score for women was about 10 points lower, a clinically consequential difference. Second, ablation offered significant QOL improvements over drug therapy for both women and men at 12 months, but the treatment effect sizes attenuated thereafter in both groups. Third, women and men had quantitatively comparable improvements in incremental QOL benefit from ablation, but these benefits over drug therapy were primarily accrued by those with worse baseline QOL. Finally, among ablation‐treated patients, absolute sex differences in QOL, as reflected in mean AFEQT and MAFSI scores, persisted over the CABANA follow‐up, suggesting that lower rates of use of ablation in women is not a sufficient primary explanation for previously reported worse QOL in women with AF. Furthermore, CABANA suggests that increased use of ablation alone will not provide women parity with men in AF‐related QOL.

The CABANA trial, which enrolled >800 women, provides one of the largest randomized comparisons of AF management strategies in women to date and offers a unique opportunity to examine sex‐specific QOL outcomes related to AF management. Consistent with prior work, baseline QOL for women with AF in CABANA was notably worse than for the men enrolled in the trial, both in terms of symptom burden and AF‐related QOL. These findings are consistent with prior observational work.^17^, ^18^ For example, Piccini and colleagues reported QOL among patients with AF in ORBIT‐AF (Outcomes Registry for Better Informed Treatment of Atrial Fibrillation), and found that the median baseline QOL as measured by the AFEQT summary score among women with AF was 80 (interquartile range [IQR], 62–92), which was significantly lower than in the subgroup of men (83; IQR, 64–94; *P*<0.001), and this relationship was consistent across the AFEQT domains and unchanged after adjustment for sociodemographic variables.^2^ Relative to the ORBIT‐AF registry findings, CABANA patients were substantially more symptomatic from their AF at baseline, with a larger sex‐related gap in AFEQT scores.

The observation that women and men with AF differ in QOL outcomes builds on >20 years of clinical investigations into AF sex‐related differences in presentation, treatment, and outcomes.^19^ One of the challenges involved in comparing AF outcomes by sex comes from the many ways in which the cohorts differ, both in standard baseline characteristics (Table S1) as well as in more subtle aspects of their AF phenotype. A recent analysis from the ORBIT‐AF registry, for example, identified 4 distinct AF phenotype clusters with significant sex‐related variations.^20^ Women constituted about half (51%) of the low comorbidity cluster but were less prevalent in the atherosclerotic comorbid cluster (31%) and the younger/behavioral disorder cluster (27%). The relevance of such clinical AF phenotyping for defining different QOL outcome trajectories remains uncertain.

In the CABANA cohort, ablation offered significant QOL improvement over drug therapy for all participants at 12 months; this assessment was a major prespecified secondary end point of the CABANA trial. In that overall intention‐to‐treat analysis, there was significant benefit of ablation over drug therapy, which did not appear to differ by sex in post hoc analysis.^10^ However, in the present analysis, we were interested in a more nuanced evaluation of QOL outcomes in women, with particular attention paid to identifying those women most likely to benefit (or not) from ablation. At the primary 12‐month postrandomization assessment of intention‐to‐treat groups, the average AFEQT summary score in women in the ablation group was clinically and statistically significantly greater than the drug therapy group (adjusted mean difference, 6.1 [95% CI, 3.5–8.6]). The difference remained significant at 24 months, but thereafter, the difference was attenuated. This pattern was seen in the overall CABANA cohort as well, and the attenuation of the treatment effect size is clearly not because of late deterioration in QOL scores for ablation patients, but rather attributable to improvements in the drug therapy arm. In a per‐protocol analysis of the overall cohort, increased crossovers from the drug therapy arm to the ablation arm largely explained this attenuation.^10^ Crossovers among women were similar to those of the overall cohort with regard to magnitude and direction, so this likely contributes to narrowed marginal benefit of ablation over drug therapy after 24 months.

When we divided patients into those with better (≥70) or worse (<70) baseline AFEQT summary scores, the mean QOL difference in AFEQT summary scores between ablation and drug therapy was larger for both women and men with lower baseline scores (Figure S4). In the women with scores ≥70, there were no differences in average QOL between ablation and drug therapy at 12 months. When examined at the patient level, those women with better baseline QOL were equally likely to achieve clinically meaningful QOL improvement in AFEQT (≥5 points) regardless of treatment assignment. In contrast, for women with baseline AFEQT <70, a clinically meaningful improvement was substantially more likely with ablation. For men, the largest proportion of ablation patients improving at 12 months by 5 or more AFEQT points came from those with baseline scores between 70 and 89. Clinically significant improvements of 5 or more points were least frequent among patients with a baseline score of 90 or above regardless of sex, a finding at least partially explained by a ceiling effect. These different treatment benefit patterns by sex as a function of baseline AFEQT may reflect the sex‐related 10‐point difference in mean baseline scores in women versus men. In other words, it appears that the patterns of scores are the same for women and men if one includes a calibration effect for the baseline score differences. Although the theoretical ceiling for AFEQT is 100, the attainable ceiling for individual patients may be lower than that perfect score and it is possible that women may encounter a ceiling effect at a lower absolute AFEQT score than men. Although AFEQT is designed to be disease/condition specific, no QOL instrument can accurately extract all the relevant effects of a specific disease from the complex background effects that also affect QOL scores, such as age and baseline health and functioning.

We also examined whether the type of AF symptoms most prominently affecting QOL differed importantly between women and men. In this context, it is relevant to note that 50% of women and 39% of men in CABANA had paroxysmal AF at trial entry.^9^ At baseline, both sexes identified the same 4 most common symptoms based on the MAFSI questionnaire: tired/lack of energy, unable to exercise, shortness of breath, and palpitations. At 12 months and the end of follow‐up, each of these 4 symptoms was more frequently reported in the sex‐stratified drug therapy groups compared with the ablation groups, and more frequently among women than men overall. Thus, although the frequency of AF symptoms was greater among women before and after treatment, the benefit of ablation at the level of specific symptoms was similar for both sexes. In light of this well‐established observation of lower QOL among women with AF and the superiority of ablation over other rhythm control strategies at suppressing AF,^3^ it remains unclear why women are less likely to undergo AF ablation despite similar lifetime prevalence of AF in men and women.^21^

### Limitations

Our study has several limitations. First, as in any unmasked procedure‐based interventional trial, knowledge of the treatment assignment may introduce biases that affect both what patients report in their QOL surveys and what their managing clinicians choose to do in response to their clinical course during trial follow‐up. Second, the repeated measures nature of QOL assessment together generates a large number of potential outcome comparisons, and a sex subgroup analysis effectively doubles that number. As reported previously, we addressed this issue for the trial overall by prespecifying 2 coprimary QOL measures (AFEQT and MAFSI) and 1 primary time point (12 months) with all other comparisons considered secondary and supportive.^10^ We also focused on treatment‐effect size estimates and their precision and consistency over time to interpret our findings and avoided using *P* values to determine which comparisons were or were not noteworthy. Third, identifying interpretive benchmarks for a clinically meaningful change in QOL scales is challenging, in as much as no gold standard exists. Using the European Heart Rhythm Association class assessed by physicians at baseline and 1 year in the ORBIT‐AF registry, Holmes and colleagues reported that a 1‐class European Heart Rhythm Association change (the clinically meaningful anchor used) corresponded to about a 5‐point change in AFEQT for individual patients.^13^ This benchmark is not directly relevant to interpreting changes at the cohort level, because the overall mean difference is a function of the mixture of different response magnitudes.^13^, ^15^ An earlier study in a smaller cohort using a single 3‐month recall‐based assessment of clinically meaningful change as the anchor suggested that approximately 20 AFEQT points was the threshold for significant changes, assessed either by the patient or their clinician.^22^ This amounts to about 1 SD of the AFEQT score distribution and is significantly larger than the result from Holmes and colleagues. Differences in the type of reference anchor used, as well as the lack of separate anchor status assessments at baseline and follow‐up, may explain why only larger changes in AFEQT were recognized as meaningful in this initial work. Finally, our study used data on binary sex (male or female) from the clinical case report form. We do not have information on self‐reported sex or gender identification.

## Conclusions

On average, women with AF in CABANA consistently reported moderate‐to severe AF‐related baseline impairments in QOL with lower baseline scores than those reported on average by men. Following randomized treatment with ablation or drug‐based therapy, QOL improvements were observed in both sexes for both treatment arms, with greater average benefit reported by those assigned to an ablation‐based strategy. The overall treatment benefit effect size was largely driven by the treatment responses of those with poorer baseline QOL. Despite clinically effective ablation, however, women averaged persistently lower QOL scores than men despite a similar relative benefit from ablation and a similar distribution of symptoms.

## Sources of Funding

This work was supported by the National Institutes of Health grants U01HL089709, U01HL089786, U01HL089907, and U01HL089645. The content of this article does not necessarily represent the views of the National Heart, Lung, and Blood Institute or the Department of Health and Human Services, St. Jude Drug Foundation and Corporation, Biosense Webster, Medtronic, or Boston Scientific Corporation.

## Disclosures

Dr Zeitler reports research support from Biosense Webster and Sanofi; research grant from Boston Scientific; consulting for Biosense Webster, Boston Scientific, and Medtronic; travel support from Medtronic. Dr Russo reports research trials funding to the hospital (Boston Scientific, BMS‐Pfizer, Medilynx, Medtronic); consulting (Abbott, Atricure, Biosense Webster, Boston Scientific, Medtronic, PaceMate); honoraria/speaking (Biotronik, BMS/Pfizer, Medtronic, Sanofi). Dr Poole reports research grants paid directly to the University of Washington from Biotonik, Kestra Medical, and AtriCure. Dr Al‐Khalidi reports grants from the National Institutes of Health/National Heart, Lung, and Blood Institute and Mayo Clinic during the conduct of the study. Dr Lee reports grants from the National Institutes of Health/National Heart, Lung, and Blood Institute and Mayo Clinic; data and safety monitoring board service on studies funded by Astra‐Zeneca, Medtronic, Merck, Amgen, and the Cardiovascular Research Foundation during the conduct of the study. Dr Bahnson reports grants from the National Institutes of Health/National Heart, Lung, and Blood Institute and Mayo Clinic during the conduct of the study; grants from St. Jude Medica, Abbott Medical, Biosense Webster, Johnson & Johnson, and Boston Scientific Corp; consulting from Cardiofocus outside of the submitted work. Dr Packer in the past 12 months has provided consulting services for Abbott, AtriFix, Biosense Webster, Cardio Syntax, EBAmed, Johnson & Johnson, MediaSphere Medical, MedLumics, Medtronic, NeuCures, St. Jude Medical, Siemens, Spectrum Dynamics, Centrix, Thermedical, and Xenter. Dr Packer received no personal compensation for these consulting activities, unless noted. Dr Packer receives research funding from Abbott, Biosense Webster, Boston Scientific/EPT, CardioInsight, EBAmed, Medtronic, NeuCures, Siemens, St. Jude Medical, Thermedical, National Institutes of Health, Robertson Foundation, Vital Project Funds, Xenter, Mr and Mrs J. Michael Cook/Fund. The Mayo Clinic and Dr Packer have a financial interest in Analyze‐AVW technology that may have been used to analyze some of the heart images in this research. In accordance with the Bayh‐Dole Act, this technology has been licensed to commercial entities, and both Mayo Clinic and Dr Packer have received royalties >$10 000, the federal threshold for significant financial interest. In addition, Mayo Clinic holds an equity position in the company to which the AVW technology has been licensed. Dr Packer and Mayo Clinic jointly have equity in a privately held company, EBAmed; and received royalties from John Wiley & Sons, Oxford, and St. Jude Medical. Dr Packer has a licensing agreement with the American Heart Association for the MAFSI survey and has contractual rights to receive future royalties under this license. Dr Mark reports grants from the National Institutes of Health/National Heart, Lung, and Blood Institute and Mayo Clinic during the conduct of the study; grants from Merck and HeartFlow; and personal fees from Novartis outside the submitted work. The remaining authors have no disclosures to report.

## Supporting information

Data S1
Tables S1–S6
Figures S1–S5
