# Supplementary material for: Effects of Ablation Versus Drug Therapy on Quality of Life by Sex in Atrial Fibrillation: Results From the CABANA Trial
Source: J Am Heart Assoc. 2023 Jan 23;12(3):e027871. doi: 10.1161/JAHA.122.027871 (PMC9973617; doi:10.1161/JAHA.122.027871)

SUPPLEMENTAL MATERIAL

Principal CABANA Investigators and Institutions
(Listed in descending order of the number of randomized patients)

Evgeny Pokushalov, Alexander Romanov, Research Institute of Circulation Pathology / Novosibirsk, Russia (147); T. Jared Bunch, Intermountain Medical Center / Murray, UT (139); Tristram Bahnson, Duke University Medical Center / Durham, NC (133); Georg Noelker, Herz-und Diabeteszentrum NRW / Bad Oeynhausen, Germany (98); Douglas Packer, Mayo Clinic / Rochester, MN (93); Gerhard Hindricks, Herzzentrum Leipzig / Leipzig, Germany (88); Andrey Ardashev, Clinical Hospital # 83 under the Federal Medical and Biological Agency / Moscow, Russia (66); Amiran Revishvili, George Matsonashvili, Bakoulev Scientific Center for Cardiovascular Surgery / Moscow, Russia (52); Pugazhendhi Vijayaraman, Geisinger Wyoming Valley Medical Center / Wilkes-Barre, PA (43); Huseyin Ince, Universitat Rostock / Rostock, Germany (previously Dietmar Baensch) (42); Christopher Piorkowski, Technische Universitat Dresden / Dresden, Germany (41); Thomas Neumann, Kerckhoff Klinik / Bad Nauheim, Germany (40); George Veenhuyzen, University of Calgary / Calgary, AB (39); Anil Gehi, University of North Carolina at Chapel Hill / Chapel Hill, NC (previously Paul Mounsey) (38); David Wilber, Loyola University Medical Center / Maywood, IL (36); Felix Sogade, Georgia Arrhythmia Consultants / Macon, GA (34); Carlo Pappone, Policlinico San Donato Center of Clinical Arrhythmia and Electrophysiology / San Donato Milanese, Italy (previously Riccardo Cappato) (32); Adam Berman, Georgia Regents University / Augusta, GA (31); Alaa Shalaby, V.A. Pittsburgh Healthcare System / Pittsburgh, PA (25); Karl-Heinz Kuck, Asklepios Klinik Saint George / Hamburg, Germany (25); Blair Halperin, Providence Saint Vincent Medical Center / Portland, OR (25); Venkat Tholakanahalli, Minneapolis V.A. Medical Center / Minneapolis, MN (24); Eugen Palma, Montefiore Medical Center / New York, NY (24); John Holshouser, The Sanger Clinic, PA / Charlotte, NC (24); Nitish Badhwar, University of California at San Francisco Medical Center / San Francisco, CA (23); Haroon Rashid, Virginia Hospital Center - Arlington / Arlington, VA (23); Craig Cameron, Oklahoma Heart Institute / Tulsa, OK (22); John Hummel, The Ohio State University Medical Center / Columbus, OH (22); Pablo Saavedra, Vanderbilt University Medical Center / Nashville, TN (previously Dawood Darbar) (21); J. Brian Deville, The Heart Hospital Baylor Plano / Plano, TX (21); Julian Chun, CCB - Cardioaniologisches Centrum Bethanien / Frankfurt, Germany (20); Javier Roman-Gonzalez, South Texas Cardiovascular Consultants / San Antonio, TX (20); Stephen Willems, Universitares Herzzentrum Hamburg / Hamburg, Germany (20); Hasan Garan, Columbia University Medical Center / New York, NY (20); Eric Michael Crespo, Hartford Hospital / Hartford, CT (19); Peter Cheung, Scott and White Memorial Hospital / Temple, TX (previously David Fitzgerald) (18); Gerian Groenefeld, Asklepios Klinik Barmbek / Hamburg, Germany (17); Claudio Schuger, Henry Ford Hospital / Detroit, MI (16); Tariq Salam, Cardiac Study Center / Tacoma, WA (16); Yanzong Yang, The First Affiliated Hospital of Dalian Medical University / Dalian, China (15); Carlo Pappone, Maria Cecilia Hospital / Cotignola, Italy (15); Dan Wichterle, Charles University / Prague 2, Czech Republic (15); Johannes Brachmann, Klinikum Coburg / Coburg, Germany (15); Josef Kautzner, Clinic of Cardiology IKEM Medical Institute / Prague 4, Czech Republic (15); John Jayachandran, Baylor All Saints Medical Center / Fort Worth, TX (15); Young-Hoon Kim, Korea University Anam Hospital / Seoul,

Korea (14); Christopher Cole, Penrose Saint Francis Health Services / Colorado Springs, CO (14); Bengt Herweg, University of South Florida / Tampa, FL (13); Martin Lowe, The Heart Hospital / London, United Kingdom (12); Anne Dougherty, University of Texas Health Science Center at Houston / Houston, TX (previously Bharat Kantharia, Nada Memon) (12); Sergey Popov, Scientific Research Institute of Cardiology of Siberian Dept. of Russian Academy of Medical Sciences / Tomsk, Russia (11); Martin Lowe, Saint Bartholomew's Hospital / London, United Kingdom (previously Richard Schilling) (11); Stefan Spitzer, Praxisklinik Herz and GefaBe / Dresden, Germany (11); Robert Bernstein, Sentara Norfolk General Hospital / Norfolk, VA (11); Jay Simonson, Park Nicollet Heart and Vascular Center/Methodist Hospital / St. Louis Park, MN (11); Eric Buch, University of California Los Angeles / Los Angeles, CA (10); Shulin Wu, Guangdong Provincial People's Hospital / Guangzhou, China (10); Mohammed Khan, Alexian Brothers Medical Center / Elk Grove Village, IL (10); Timothy Shinn, Saint Joseph Mercy Hospital / Ann Arbor, MI (previously James Kappler) (10); Petr Neuzil, Na Homolce Hospital / Prague, Czech Republic (9); James Mangrum, University of Virginia Health System / Charlottesville, VA (9); Hugh Calkins, Johns Hopkins Hospital / Baltimore, MD (9); Mario Gonzalez, Penn State University Cardiovascular Center / Hershey, PA (9); Moussa Mansour, Massachusetts General Hospital / Boston, MA (9); Markus Zabel, Georg-August-University / Goettingen, Germany (8); Jonathan Kalman, Royal Melbourne Hospital / Parkville, Australia (8); Jose Sanchez, Saint John's Mercy Heart Hospital / St. Louis, MO (8); Steven Rothman, Lankenau Medical Center / Wynnewood, PA (8); Anil Bhandari, Good Samaritan Hospital / Los Angeles, CA (8); Cynthia Tracy, George Washington University Medical Center / Washington, DC (8); Raul Mitrani, University of Miami Health System / Miami, FL (7); Vicken Vorperian, Waukesha Memorial Hospital / Waukesha, WI (7); Derek Connelly, Golden Jubilee Hospital / Glasgow, United Kingdom (7); Darryl Wells, Swedish Medical Center - Providence Campus / Seattle, WA (7); Chang-Sheng Ma , Beijing Anzhen Hospital / Beijing, China (7); Atul Verma, Southlake Regional Health Centre / Newmarket, ON (7); S. Luke Kusmirek, Drexel University College of Medicine / Philadelphia, PA (7); Melissa Robinson, University of Washington Medical Center / Seattle, WA (previously Robert Rho, Mohan Viswanathan) (7); Donald Rubenstein, Greenville Hospital System University Medical Center / Greenville, SC (6); Emilio Vanoli, Policlinico Multimedical Cardiology and Arrhythmia Centre / Milan, Italy (previously Annibale Montenero) (6); Shu Zhang, Fuwai Hospital / Beijing, China (6); Jennifer Cummings, The University of Toledo / Toledo, OH (previously Mohammed Kanjwal) (6); Mohan Viswanathan, Stanford University Medical Center / Stanford, CA (previously Amin Al Ahmad, Paul Zei) (6); George Monir, Florida Hospital / Orlando, FL (6); Francis Marchlinski, University of Pennsylvania Health System / Philadelphia, PA (6); Jay Franklin, Baylor Heart and Vascular Hospital / Dallas, TX (previously Robert Kowal) (6); Bruce Koplan, Brigham and Women's Hospital / Boston, MA (previously Gregory Michaud) (6); Prashanthan Sanders, Royal Adelaide Hospital / Adelaide, Australia (5); Eric Rashba, Stony Brook University Medical Center / Stony Brook, NY (5); Mark Gallagher, Saint George's Hospital Medical School / London, United Kingdom (5); Bernd Gonska, Saint Vincentius-Kliniken / Karlsruhe, Germany (5); Minglong Chen, First Affiliated Hospital of Nanjing Medical University / Nanjing, China (5); Peter Leong-Sit, University of Western Ontario - London Health Sciences Centre / London, Ontario (5); John Zimmerman,

Hackensack University Medical Center / Hackensack, NJ (5); Nayer Pezeshkian, University of California Davis Medical Center / Sacramento, CA (5); Andrew Cohen, The Medical Center of Aurora / Aurora, CO (5); Saulius Kalvaitis, Saint Louis Heart and Vascular / St. Louis, MO (4); David Davies, Saint Mary's Hospital / London, United Kingdom (4); Martin Borggrefe, University Hospital of Mannheim / Mannheim, Germany (4); Hui-Nam Pak, Yonsei University Severance Hospital / Seoul, Korea (4); Andrea Russo, Cooper University Hospital / Camden, NJ (4); Charles Henrikson, Oregon Health and Science University / Portland, OR (previously Jack Kron) (4); Gerald Greer, Arkansas Cardiology, PA / Little Rock, AR (4); James Coromilas, Robert Wood Johnson University Hospital / New Brunswick, NJ (3); Farhat Khairallah, Tallahassee Memorial Hospital / Tallahassee, FL (3); Guillermo Sosa-Suarez, Albany Associates in Cardiology / Albany, NY (3); Bruce Lindsay, Cleveland Clinic Foundation / Cleveland, OH (3); Westby Fisher, North Shore University Health System/Evanston Hospital / Evanston, IL (3); Steven Bailin, Mercy Medical Center / Des Moines, IA (3); Andy Tran, Scottsdale Healthcare / Scottsdale, AZ (2); Zdenek Starek, Saint Anne's University Hospital, ICRC / Brno, Czech Republic (2); Mark Preminger, The Valley Hospital / Ridgewood, NJ (2); Robert Sheppard, Northside Hospital and Heart Institute / St. Petersburg, FL (2); Alexandru Costea, University of Cincinnati Medical Center / Cincinnati, OH (2); Kenneth Ellenbogen, Virginia Commonwealth University Medical Center / Richmond, VA (2); Thomas Arentz, Herz-Zentrum Bad Krozingen / Bad Krozingen, Germany (1); Roberto De Ponti, Ospedale di Circolo e Fondazione Macchi / Varese, Italy (1); Ryan Aleong, University of Colorado Hospital / Aurora, CO (1); Byron Colley III, Jackson Heart Clinic / Jackson, MS (1); Khawaja Baig, Medisync / Cincinnati, OH (1); Kousik Krishnan, Rush University Medical Center / Chicago, IL (1); Syamkumar Divakara Menon, Hamilton Health Sciences / Hamilton, ON (previously Carlos Morillo) (1); Tony Simmons, Wake Forest University Health Sciences / Winston Salem, NC (1); Gregory Bruce, Memorial Health Care System / Chattanooga, TN (1); Larry Chinitz, Tisch Hospital (New York University Langone Medical Center) / New York, NY (1); Andrea Natale, Texas Cardiac Arrhythmia / Austin, TX (1); Riccardo Cappato, IRCCS Istituto Clinico Humanitas / Milano, Italy.

Table S1. Baseline characteristics by sex

	**Men (N=1385)**	**Women (N=819)**	**p-value**
Age			<.0001
Median (Q1, Q3)	66.5 (60.1, 70.9)	69.4 (65.1, 73.7)	
N	1385	819	
Age – categories			<.0001
<65 yrs	568/1385 (41.0%)	198/819 (24.2%)	
≥ 65 to <75 yrs	662/1385 (47.8%)	468/819 (57.1%)	
≥ 75 yrs	155/1385 (11.2%)	153/819 (18.7%)	
Race			0.0520
White	1259/1384 (91.0%)	766/816 (93.9%)	
Black or African American	55/1384 (4.0%)	22/816 (2.7%)	
Other	70/1384 (5.1%)	28/816 (3.4%)	
Minority: Hispanic or non-White	158/1381 (11.4%)	67/817 (8.2%)	0.0154
BMI(kg/m2)			0.8296
Median (Q1, Q3)	30.0 (26.6, 34.3)	30.1 (26.1, 35.2)	
N	1370	800	
AF Severity (CCS Class)			<.0001
Class 0	160/1375 (11.6%)	63/816 (7.7%)	
Class 1	246/1375 (17.9%)	93/816 (11.4%)	
Class 2	433/1375 (31.5%)	270/816 (33.1%)	
Class 3	457/1375 (33.2%)	326/816 (40.0%)	
Class 4	79/1375 (5.7%)	64/816 (7.8%)	
Heart function severity (NYHA Class) ≥ II	433/1373 (31.5%)	345/813 (42.4%)	<.0001
**Medical History**			
Hypertension (>140/90 mmHg)	1100/1385 (79.4%)	676/818 (82.6%)	0.0649
Baseline left ventricular hypertrophy	444/1025 (43.3%)	218/619 (35.2%)	0.0012
Diabetes (Glucose ≥126 mg/dl)	360/1385 (26.0%)	201/818 (24.6%)	0.4596
Prior CVA or TIA	129/1385 (9.3%)	91/818 (11.1%)	0.1708
Coronary artery disease	332/1385 (24.0%)	92/818 (11.2%)	<.0001
Sleep apnea	372/1385 (26.9%)	136/818 (16.6%)	<.0001
Left ventricular ejection fraction ≤ 35	58/948 (6.1%)	11/582 (1.9%)	0.0001

	Men (N=1385)	Women (N=819)	p-value
**Co-morbidities**			
CHA_2DS_2-VASc Score			<.0001
Median (Q1, Q3)	2.0 (1.0, 3.0)	3.0 (3.0, 4.0)	
N	1385	819	
**Arrhythmia History**			
Years since first onset of AF			0.4818
Median (Q1, Q3)	1.0 (0.3, 3.9)	1.2 (0.3, 3.8)	
N	1373	812	
**Type of atrial fibrillation**			<.0001
Paroxysmal	540/1385 (39.0%)	406/818 (49.6%)	
Persistent	690/1385 (49.8%)	352/818 (43.0%)	
Long-standing persistent	155/1385 (11.2%)	60/818 (7.3%)	
Prior direct current cardioversion of AF	547/1384 (39.5%)	262/818 (32.0%)	0.0004
Current or past use of Rhythm Control Therapy reported at time of enrollment	638/1312 (48.6%)	400/782 (51.2%)	0.2640
≥2 Rhythm Control Drugs	109/638 (17.1%)	79/400 (19.8%)	0.2778
Crossover	263/1385 (19.0%)	140/819 (17.1%)	0.2660
Study Withdrawal	138/1385 (10.0%)	101/819 (12.3%)	0.0840
Follow-up time			0.0136
Median (Q1, Q3)	49.7 (30.4, 62.5)	47.3 (28.7, 61.4)	
N	1385	819	
Modified from Russo et al.[9]			

BMI = Body Mass Index, AF = atrial fibrillation, CCS = Canadian Cardiovascular Society, NYHA = New York Heart Association, CVA = Cerebrovascular accident, TIA = transient ischemic attack, CHA_2DS_2-VASc = AF-related stroke risk score combining Congestive heart failure, Hypertension, Age > 75, Diabetes mellitus, history of Stroke/TIA, Vascular disease, Age > 65, Sex category.

Table S2. Patient characteristics of enrolled women by baseline AFEQT Summary Score and treatment assignment

	Catheter Ablation (N=413)		Drug Therapy (N=406)	
Characteristics	Baseline AFEQT Summary Score <70 (N=296)	Baseline AFEQT Summary Score ≥**70** (N=112)	Baseline AFEQT Summary Score <70 (N=306)	Baseline AFEQT Summary Score ≥**70** (N=94)
Age				
Median (Q1, Q3)	69.1 (65.2, 73.6)	70.5 (65.6, 74.7)	68.7 (64.4, 73.1)	71.0 (66.1, 74.3)
N	296	112	306	94
Age – categories, years				
<65	68/296 (23.0%)	24/112 (21.4%)	86/306 (28.1%)	17/94 (18.1%)
65 to 74	172/296 (58.1%)	61/112 (54.5%)	171/306 (55.9%)	59/94 (62.8%)
≥75	56/296 (18.9%)	27/112 (24.1%)	49/306 (16.0%)	18/94 (19.1%)
Race				
White	282/296 (95.3%)	102/112 (91.1%)	284/305 (93.1%)	88/93 (94.6%)
Black or African American	6/296 (2.0%)	2/112 (1.8%)	11/305 (3.6%)	3/93 (3.2%)
Other	8/296 (2.7%)	8/112 (7.1%)	10/305 (3.3%)	2/93 (2.2%)
Hispanic or non-White	19/296 (6.4%)	12/111 (10.8%)	29/306 (9.5%)	6/94 (6.4%)
BMI (kg/m2)				
Median (Q1, Q3)	30.8 (26.7, 35.5)	28.1 (25.5, 32.4)	30.5 (26.4, 36.0)	29.2 (24.6, 34.4)
N	287	109	303	91
AF Severity (CCS Class)				
Class 0	19/295 (6.4%)	12/112 (10.7%)	22/306 (7.2%)	8/93 (8.6%)
Class 1	28/295 (9.5%)	14/112 (12.5%)	34/306 (11.1%)	16/93 (17.2%)

Class 2	98/295 (33.2%)	41/112 (36.6%)	93/306 (30.4%)	37/93 (39.8%)
Class 3	119/295 (40.3%)	41/112 (36.6%)	130/306 (42.5%)	31/93 (33.3%)
Class 4	31/295 (10.5%)	4/112 (3.6%)	27/306 (8.8%)	1/93 (1.1%)
Heart function severity (NYHA Class) ≥II	134/294 (45.6%)	35/112 (31.3%)	136/304 (44.7%)	37/93 (39.8%)
Medical History				
Hypertension (>140/90 mmHg)	242/296 (81.8%)	91/112 (81.3%)	256/306 (83.7%)	77/94 (81.9%)
Baseline left ventricular hypertrophy	88/240 (36.7%)	22/82 (26.8%)	86/223 (38.6%)	18/68 (26.5%)
Diabetes (Glucose ≥126 mg/dl)	72/296 (24.3%)	27/112 (24.1%)	79/306 (25.8%)	19/94 (20.2%)
Prior CVA or TIA	35/296 (11.8%)	16/112 (14.3%)	30/306 (9.8%)	9/94 (9.6%)
Coronary artery disease	34/296 (11.5%)	14/112 (12.5%)	35/306 (11.4%)	9/94 (9.6%)
Sleep apnea	52/296 (17.6%)	21/112 (18.8%)	51/306 (16.7%)	12/94 (12.8%)
Left ventricular ejection fraction ≤35%	3/223 (1.3%)	2/79 (2.5%)	5/211 (2.4%)	1/63 (1.6%)
CHADS-VASc Score				
Median (Q1, Q3)	3.0 (3.0, 4.0)	4.0 (3.0, 4.0)	3.0 (3.0, 4.0)	3.0 (3.0, 4.0)
N	296	112	306	94
Arrhythmia History				
Years since first onset of AF				
Median (Q1, Q3)	1.1 (0.2, 3.8)	1.1 (0.3, 4.2)	1.5 (0.3, 4.0)	1.0 (0.4, 3.5)
N	294	111	303	94
Type of atrial fibrillation				
Paroxysmal	123/296 (41.6%)	68/112 (60.7%)	169/306 (55.2%)	39/94 (41.5%)
Persistent	150/296 (50.7%)	34/112 (30.4%)	121/306 (39.5%)	44/94 (46.8%)
Long-standing persistent	23/296 (7.8%)	10/112 (8.9%)	16/306 (5.2%)	11/94 (11.7%)
Prior direct current cardioversion of AF	106/296 (35.8%)	23/112 (20.5%)	98/306 (32.0%)	32/94 (34.0%)

Current or past use of rhythm control therapy reported at time of enrollment	141/282 (50.0%)	45/109 (41.3%)	160/293 (54.6%)	52/90 (57.8%)
≥2 rhythm control drugs	36/141 (25.5%)	11/45 (24.4%)	25/160 (15.6%)	7/52 (13.5%)
Crossover	26/296 (8.8%)	12/112 (10.7%)	79/306 (25.8%)	21/94 (22.3%)
Follow-up time, Median (Q1, Q3)	48.1 (29.2, 61.2)	47.5 (27.0, 63.5)	46.8 (29.5, 61.7)	42.6 (30.0, 61.7)

BMI = Body Mass Index, AF = atrial fibrillation, CCS = Canadian Cardiovascular Society, NYHA = New York Heart Association, CVA = Cerebrovascular accident, TIA = transient ischemic attack, CHA_2DS_2-VASc = AF-related stroke risk score combining Congestive heart failure, Hypertension, Age > 75, Diabetes mellitus, history of Stroke/TIA, Vascular disease, Age > 65, Sex category.

Table S3. Baseline characteristics among men by treatment and baseline AFEQT summary score (<70 vs 70+)

	Catheter Ablation (N=695)		Drug Therapy (N=690)	
**Characteristics**	**Baseline AFEQT summary score < 70 (N=356)**	**Baseline AFEQT summary score ≥70 (N=320)**	**Baseline AFEQT summary score < 70 (N=344)**	**Baseline AFEQT summary score ≥70 (N=334)**
Age				
Median (Q1, Q3)	66.3 (59.4, 71.0)	67.3 (60.9, 71.3)	66.1 (60.8, 70.5)	67.0 (60.9, 71.9)
N	356	320	344	334
Age - categories, years				
<65	153/356 (43.0%)	120/320 (37.5%)	145/344 (42.2%)	135/334 (40.4%)
65 to 74	164/356 (46.1%)	168/320 (52.5%)	161/344 (46.8%)	156/334 (46.7%)
≥75	39/356 (11.0%)	32/320 (10.0%)	38/344 (11.0%)	43/334 (12.9%)
Race				
White	314/355 (88.5%)	299/320 (93.4%)	308/344 (89.5%)	314/334 (94.0%)
Black or African American	21/355 (5.9%)	9/320 (2.8%)	14/344 (4.1%)	7/334 (2.1%)
Other	20/355 (5.6%)	12/320 (3.8%)	22/344 (6.4%)	13/334 (3.9%)
Hispanic or non-White	53/354 (15.0%)	25/319 (7.8%)	44/344 (12.8%)	29/333 (8.7%)
BMI (kg/m2)				
Median (Q1, Q3)	30.4 (26.5, 34.6)	29.7 (26.9, 33.5)	30.2 (26.6, 35.4)	30.1 (26.9, 34.0)
N	349	317	342	331
AF Severity (CCS Class)				
Class 0	22/353 (6.2%)	47/317 (14.8%)	28/343 (8.2%)	58/333 (17.4%)
Class 1	39/353 (11.0%)	81/317 (25.6%)	36/343 (10.5%)	82/333 (24.6%)
Class 2	108/353 (30.6%)	99/317 (31.2%)	102/343 (29.7%)	116/333 (34.8%)
Class 3	153/353 (43.3%)	79/317 (24.9%)	146/343 (42.6%)	71/333 (21.3%)
Class 4	31/353 (8.8%)	11/317 (3.5%)	31/343 (9.0%)	6/333 (1.8%)
Heart function severity (NYHA Class) ≥II	128/351 (36.5%)	74/316 (23.4%)	141/342 (41.2%)	80/333 (24.0%)

Medical History				
Hypertension (>140/90 mmHg)	281/356 (78.9%)	243/320 (75.9%)	274/344 (79.7%)	277/334 (82.9%)
Baseline left ventricular hypertrophy	119/283 (42.0%)	99/241 (41.1%)	113/242 (46.7%)	106/236 (44.9%)
Diabetes (Glucose ≥126 mg/dl)	97/356 (27.2%)	74/320 (23.1%)	91/344 (26.5%)	88/334 (26.3%)
Prior CVA or TIA	34/356 (9.6%)	29/320 (9.1%)	40/344 (11.6%)	23/334 (6.9%)
Coronary artery disease	96/356 (27.0%)	61/320 (19.1%)	104/344 (30.2%)	65/334 (19.5%)
Sleep apnea	96/356 (27.0%)	90/320 (28.1%)	105/344 (30.5%)	77/334 (23.1%)
Left ventricular ejection fraction ≤35	20/253 (7.9%)	12/220 (5.5%)	16/228 (7.0%)	9/229 (3.9%)
CHADS-VASc Score				
Median (Q1, Q3)	2.0 (1.0, 3.0)	2.0 (1.0, 3.0)	2.0 (2.0, 3.0)	2.0 (1.0, 3.0)
N	356	320	344	334
Arrhythmia History				
Years since first onset of AF				
Median (Q1, Q3)	0.9 (0.2, 3.8)	1.1 (0.3, 4.1)	1.2 (0.3, 4.5)	0.8 (0.3, 2.9)
N	354	318	339	332
Type of atrial fibrillation				
Paroxysmal	156/356 (43.8%)	114/320 (35.6%)	139/344 (40.4%)	118/334 (35.3%)
Persistent	163/356 (45.8%)	165/320 (51.6%)	171/344 (49.7%)	177/334 (53.0%)
Long-standing persistent	37/356 (10.4%)	41/320 (12.8%)	34/344 (9.9%)	39/334 (11.7%)
Prior direct current cardioversion of AF	125/356 (35.1%)	132/319 (41.4%)	147/344 (42.7%)	132/334 (39.5%)
Current or past use of rhythm control therapy reported at time of enrollment	155/326 (47.5%)	133/307 (43.3%)	163/325 (50.2%)	167/326 (51.2%)
≥2 rhythm control drugs	21/155 (13.5%)	19/133 (14.3%)	34/163 (20.9%)	28/167 (16.8%)
Crossover	28/356 (7.9%)	27/320 (8.4%)	114/344 (33.1%)	84/334 (25.1%)
Follow-up time, Median (Q1, Q3)	49.1 (30.7, 62.5)	51.3 (31.9, 61.6)	51.5 (31.9, 63.4)	49.0 (29.7, 63.9)

BMI = Body Mass Index, AF = atrial fibrillation, CCS = Canadian Cardiovascular Society, NYHA = New York Heart Association, CVA = Cerebrovascular accident, TIA = transient ischemic attack, CHA_2DS_2-VASc = AF-related stroke risk score combining Congestive heart failure, Hypertension, Age > 75, Diabetes mellitus, history of Stroke/TIA, Vascular disease, Age > 65, Sex category.

Table S4. Frequency of MAFSI components by sex and treatment assignment

	Women			Men		
MAFSI	Catheter Ablation (N=413)	Drug Therapy (N=406)	p-value	Catheter Ablation (N=695)	Drug Therapy (N=690)	p-value
**Palpitation heart fluttering/racing**						
**Baseline**			0.3844			0.3025
Never	52/400 (13.0%)	46/395 (11.6%)		154/667 (23.1%)	176/664 (26.5%)	
Rarely	57/400 (14.3%)	42/395 (10.6%)		124/667 (18.6%)	114/664 (17.2%)	
Sometimes	138/400 (34.5%)	153/395 (38.7%)		238/667 (35.7%)	207/664 (31.2%)	
Often	119/400 (29.8%)	126/395 (31.9%)		115/667 (17.2%)	128/664 (19.3%)	
Always	34/400 (8.5%)	28/395 (7.1%)		36/667 (5.4%)	39/664 (5.9%)	
**Month 12**			0.0020			0.0811
Never	139/311 (44.7%)	97/314 (30.9%)		308/516 (59.7%)	270/516 (52.3%)	
Rarely	62/311 (19.9%)	65/314 (20.7%)		118/516 (22.9%)	121/516 (23.4%)	
Sometimes	87/311 (28.0%)	111/314 (35.4%)		67/516 (13.0%)	93/516 (18.0%)	
Often	19/311 (6.1%)	38/314 (12.1%)		20/516 (3.9%)	27/516 (5.2%)	
Always	4/311 (1.3%)	3/314 (1.0%)		3/516 (0.6%)	5/516 (1.0%)	
**Month 24**			0.0010			0.0065
Never	120/277 (43.3%)	80/264 (30.3%)		287/477 (60.2%)	236/457 (51.6%)	
Rarely	64/277 (23.1%)	65/264 (24.6%)		89/477 (18.7%)	106/457 (23.2%)	
Sometimes	68/277 (24.5%)	65/264 (24.6%)		83/477 (17.4%)	77/457 (16.8%)	
Often	20/277 (7.2%)	43/264 (16.3%)		15/477 (3.1%)	27/457 (5.9%)	
Always	5/277 (1.8%)	11/264 (4.2%)		3/477 (0.6%)	11/457 (2.4%)	
**Month 36**			0.1462			0.0057
Never	80/205 (39.0%)	82/204 (40.2%)		213/364 (58.5%)	194/354 (54.8%)	
Rarely	49/205 (23.9%)	36/204 (17.6%)		90/364 (24.7%)	67/354 (18.9%)	

Sometimes	54/205 (26.3%)	53/204 (26.0%)		49/364 (13.5%)	61/354 (17.2%)	
Often	19/205 (9.3%)	22/204 (10.8%)		8/364 (2.2%)	20/354 (5.6%)	
Always	3/205 (1.5%)	11/204 (5.4%)		4/364 (1.1%)	12/354 (3.4%)	
**Month 48**			0.0150			0.2139
Never	55/151 (36.4%)	39/140 (27.9%)		158/268 (59.0%)	148/277 (53.4%)	
Rarely	33/151 (21.9%)	46/140 (32.9%)		56/268 (20.9%)	54/277 (19.5%)	
Sometimes	46/151 (30.5%)	29/140 (20.7%)		38/268 (14.2%)	48/277 (17.3%)	
Often	16/151 (10.6%)	20/140 (14.3%)		11/268 (4.1%)	13/277 (4.7%)	
Always	1/151 (0.7%)	6/140 (4.3%)		5/268 (1.9%)	14/277 (5.1%)	
**Month 60**			0.0791			0.1186
Never	41/105 (39.0%)	36/110 (32.7%)		110/174 (63.2%)	105/183 (57.4%)	
Rarely	30/105 (28.6%)	27/110 (24.5%)		38/174 (21.8%)	37/183 (20.2%)	
Sometimes	17/105 (16.2%)	31/110 (28.2%)		23/174 (13.2%)	27/183 (14.8%)	
Often	14/105 (13.3%)	8/110 (7.3%)		2/174 (1.1%)	9/183 (4.9%)	
Always	3/105 (2.9%)	8/110 (7.3%)		1/174 (0.6%)	5/183 (2.7%)	
**Shortness of breath**						
**Baseline**			0.9239			0.8952
Never	70/399 (17.5%)	61/391 (15.6%)		165/661 (25.0%)	168/663 (25.3%)	
Rarely	62/399 (15.5%)	67/391 (17.1%)		127/661 (19.2%)	129/663 (19.5%)	
Sometimes	136/399 (34.1%)	135/391 (34.5%)		227/661 (34.3%)	221/663 (33.3%)	
Often	103/399 (25.8%)	103/391 (26.3%)		110/661 (16.6%)	119/663 (17.9%)	
Always	28/399 (7.0%)	25/391 (6.4%)		32/661 (4.8%)	26/663 (3.9%)	
**Month 12**			0.1255			<.0001
Never	121/308 (39.3%)	94/311 (30.2%)		300/514 (58.4%)	213/512 (41.6%)	
Rarely	53/308 (17.2%)	50/311 (16.1%)		93/514 (18.1%)	129/512 (25.2%)	
Sometimes	79/308 (25.6%)	101/311 (32.5%)		76/514 (14.8%)	114/512 (22.3%)	
Often	41/308 (13.3%)	48/311 (15.4%)		33/514 (6.4%)	45/512 (8.8%)	

Always	14/308 (4.5%)	18/311 (5.8%)		12/514 (2.3%)	11/512 (2.1%)	
**Month 24**			0.3025			0.0482
Never	101/278 (36.3%)	88/266 (33.1%)		264/476 (55.5%)	219/457 (47.9%)	
Rarely	56/278 (20.1%)	46/266 (17.3%)		86/476 (18.1%)	84/457 (18.4%)	
Sometimes	80/278 (28.8%)	80/266 (30.1%)		84/476 (17.6%)	101/457 (22.1%)	
Often	27/278 (9.7%)	41/266 (15.4%)		35/476 (7.4%)	36/457 (7.9%)	
Always	14/278 (5.0%)	11/266 (4.1%)		7/476 (1.5%)	17/457 (3.7%)	
**Month 36**			0.0135			0.1568
Never	71/205 (34.6%)	84/205 (41.0%)		200/362 (55.2%)	177/354 (50.0%)	
Rarely	43/205 (21.0%)	31/205 (15.1%)		68/362 (18.8%)	65/354 (18.4%)	
Sometimes	61/205 (29.8%)	48/205 (23.4%)		66/362 (18.2%)	65/354 (18.4%)	
Often	18/205 (8.8%)	36/205 (17.6%)		20/362 (5.5%)	30/354 (8.5%)	
Always	12/205 (5.9%)	6/205 (2.9%)		8/362 (2.2%)	17/354 (4.8%)	
**Month 48**			0.3018			0.8820
Never	52/151 (34.4%)	55/139 (39.6%)		147/268 (54.9%)	144/277 (52.0%)	
Rarely	25/151 (16.6%)	24/139 (17.3%)		46/268 (17.2%)	47/277 (17.0%)	
Sometimes	39/151 (25.8%)	40/139 (28.8%)		48/268 (17.9%)	51/277 (18.4%)	
Often	29/151 (19.2%)	14/139 (10.1%)		18/268 (6.7%)	25/277 (9.0%)	
Always	6/151 (4.0%)	6/139 (4.3%)		9/268 (3.4%)	10/277 (3.6%)	
**Month 60**			0.8881			0.3488
Never	41/104 (39.4%)	38/108 (35.2%)		103/171 (60.2%)	98/183 (53.6%)	
Rarely	19/104 (18.3%)	21/108 (19.4%)		35/171 (20.5%)	34/183 (18.6%)	
Sometimes	27/104 (26.0%)	27/108 (25.0%)		28/171 (16.4%)	39/183 (21.3%)	
Often	10/104 (9.6%)	15/108 (13.9%)		4/171 (2.3%)	10/183 (5.5%)	
Always	7/104 (6.7%)	7/108 (6.5%)		1/171 (0.6%)	2/183 (1.1%)	
**Unable to exercise**						
**Baseline**			0.8920			0.9165

Never	112/398 (28.1%)	102/393 (26.0%)		271/662 (40.9%)	281/661 (42.5%)	
Rarely	65/398 (16.3%)	64/393 (16.3%)		121/662 (18.3%)	112/661 (16.9%)	
Sometimes	106/398 (26.6%)	103/393 (26.2%)		134/662 (20.2%)	128/661 (19.4%)	
Often	55/398 (13.8%)	55/393 (14.0%)		70/662 (10.6%)	68/661 (10.3%)	
Always	60/398 (15.1%)	69/393 (17.6%)		66/662 (10.0%)	72/661 (10.9%)	
**Month 12**			0.0024			0.2120
Never	180/310 (58.1%)	130/309 (42.1%)		356/514 (69.3%)	319/510 (62.5%)	
Rarely	36/310 (11.6%)	51/309 (16.5%)		62/514 (12.1%)	80/510 (15.7%)	
Sometimes	48/310 (15.5%)	59/309 (19.1%)		50/514 (9.7%)	63/510 (12.4%)	
Often	21/310 (6.8%)	29/309 (9.4%)		23/514 (4.5%)	24/510 (4.7%)	
Always	25/310 (8.1%)	40/309 (12.9%)		23/514 (4.5%)	24/510 (4.7%)	
**Month 24**			0.0061			0.0684
Never	159/271 (58.7%)	126/265 (47.5%)		337/476 (70.8%)	290/453 (64.0%)	
Rarely	39/271 (14.4%)	43/265 (16.2%)		51/476 (10.7%)	71/453 (15.7%)	
Sometimes	37/271 (13.7%)	36/265 (13.6%)		44/476 (9.2%)	45/453 (9.9%)	
Often	11/271 (4.1%)	32/265 (12.1%)		21/476 (4.4%)	15/453 (3.3%)	
Always	25/271 (9.2%)	28/265 (10.6%)		23/476 (4.8%)	32/453 (7.1%)	
**Month 36**			0.6517			0.1564
Never	103/203 (50.7%)	109/204 (53.4%)		249/362 (68.8%)	222/351 (63.2%)	
Rarely	28/203 (13.8%)	30/204 (14.7%)		43/362 (11.9%)	35/351 (10.0%)	
Sometimes	35/203 (17.2%)	29/204 (14.2%)		30/362 (8.3%)	46/351 (13.1%)	
Often	15/203 (7.4%)	20/204 (9.8%)		20/362 (5.5%)	21/351 (6.0%)	
Always	22/203 (10.8%)	16/204 (7.8%)		20/362 (5.5%)	27/351 (7.7%)	
**Month 48**			0.8944			0.4953
Never	81/151 (53.6%)	72/140 (51.4%)		193/268 (72.0%)	182/278 (65.5%)	
Rarely	22/151 (14.6%)	23/140 (16.4%)		28/268 (10.4%)	35/278 (12.6%)	
Sometimes	24/151 (15.9%)	21/140 (15.0%)		18/268 (6.7%)	28/278 (10.1%)	

Often	15/151 (9.9%)	12/140 (8.6%)		13/268 (4.9%)	14/278 (5.0%)	
Always	9/151 (6.0%)	12/140 (8.6%)		16/268 (6.0%)	19/278 (6.8%)	
**Month 60**			0.6731			0.1857
Never	52/101 (51.5%)	51/110 (46.4%)		122/171 (71.3%)	116/183 (63.4%)	
Rarely	9/101 (8.9%)	17/110 (15.5%)		23/171 (13.5%)	26/183 (14.2%)	
Sometimes	18/101 (17.8%)	17/110 (15.5%)		12/171 (7.0%)	23/183 (12.6%)	
Often	10/101 (9.9%)	12/110 (10.9%)		4/171 (2.3%)	10/183 (5.5%)	
Always	12/101 (11.9%)	13/110 (11.8%)		10/171 (5.8%)	8/183 (4.4%)	
**Tired/lack of energy**						
**Baseline**			0.9126			0.8801
Never	31/397 (7.8%)	34/391 (8.7%)		95/660 (14.4%)	97/662 (14.7%)	
Rarely	63/397 (15.9%)	58/391 (14.8%)		126/660 (19.1%)	140/662 (21.1%)	
Sometimes	141/397 (35.5%)	136/391 (34.8%)		239/660 (36.2%)	225/662 (34.0%)	
Often	107/397 (27.0%)	101/391 (25.8%)		129/660 (19.5%)	130/662 (19.6%)	
Always	55/397 (13.9%)	62/391 (15.9%)		71/660 (10.8%)	70/662 (10.6%)	
**Month 12**			0.0006			0.0635
Never	90/310 (29.0%)	53/312 (17.0%)		211/512 (41.2%)	176/514 (34.2%)	
Rarely	59/310 (19.0%)	48/312 (15.4%)		118/512 (23.0%)	113/514 (22.0%)	
Sometimes	88/310 (28.4%)	118/312 (37.8%)		126/512 (24.6%)	144/514 (28.0%)	
Often	49/310 (15.8%)	52/312 (16.7%)		37/512 (7.2%)	56/514 (10.9%)	
Always	24/310 (7.7%)	41/312 (13.1%)		20/512 (3.9%)	25/514 (4.9%)	
**Month 24**			0.0894			0.0120
Never	76/278 (27.3%)	52/265 (19.6%)		209/474 (44.1%)	156/455 (34.3%)	
Rarely	56/278 (20.1%)	50/265 (18.9%)		93/474 (19.6%)	98/455 (21.5%)	
Sometimes	91/278 (32.7%)	94/265 (35.5%)		125/474 (26.4%)	129/455 (28.4%)	
Often	40/278 (14.4%)	42/265 (15.8%)		32/474 (6.8%)	47/455 (10.3%)	
Always	15/278 (5.4%)	27/265 (10.2%)		15/474 (3.2%)	25/455 (5.5%)	

**Month 36**			0.1111			0.0591
Never	55/205 (26.8%)	55/205 (26.8%)		162/363 (44.6%)	135/350 (38.6%)	
Rarely	34/205 (16.6%)	42/205 (20.5%)		83/363 (22.9%)	67/350 (19.1%)	
Sometimes	71/205 (34.6%)	51/205 (24.9%)		78/363 (21.5%)	88/350 (25.1%)	
Often	29/205 (14.1%)	44/205 (21.5%)		23/363 (6.3%)	39/350 (11.1%)	
Always	16/205 (7.8%)	13/205 (6.3%)		17/363 (4.7%)	21/350 (6.0%)	
**Month 48**			0.1764			0.4403
Never	47/151 (31.1%)	40/140 (28.6%)		132/269 (49.1%)	119/278 (42.8%)	
Rarely	24/151 (15.9%)	30/140 (21.4%)		48/269 (17.8%)	52/278 (18.7%)	
Sometimes	38/151 (25.2%)	38/140 (27.1%)		62/269 (23.0%)	70/278 (25.2%)	
Often	33/151 (21.9%)	18/140 (12.9%)		22/269 (8.2%)	26/278 (9.4%)	
Always	9/151 (6.0%)	14/140 (10.0%)		5/269 (1.9%)	11/278 (4.0%)	
**Month 60**			0.3407			0.1999
Never	32/102 (31.4%)	26/110 (23.6%)		85/172 (49.4%)	68/183 (37.2%)	
Rarely	19/102 (18.6%)	26/110 (23.6%)		36/172 (20.9%)	44/183 (24.0%)	
Sometimes	32/102 (31.4%)	28/110 (25.5%)		34/172 (19.8%)	49/183 (26.8%)	
Often	11/102 (10.8%)	20/110 (18.2%)		11/172 (6.4%)	16/183 (8.7%)	
Always	8/102 (7.8%)	10/110 (9.1%)		6/172 (3.5%)	6/183 (3.3%)	

MAFSI = Mayo Atrial Fibrillation Symptom Inventory

Table S5. AFEQT Summary Score by Baseline AFEQT Summary Score

AFEQT Summary Score, Baseline AFEQT Summary Score <70											
	Women						Men					
	Catheter Ablation (N=296)		Drug Therapy (N=306)				Catheter Ablation (N=356)		Drug Therapy (N=344)			
	No.	Mean (SD)	No.	Mean (SD)	Mean adjusted difference (CA minus drug) (95% CI)	p-value	No.	Mean (SD)	No.	Mean (SD)	Mean adjusted difference (CA minus drug) (95% CI)	p-value
Baseline	296	47.5 (14.1)	306	47.1 (13.6)	0.4 (-1.8 to 2.7)	0.694	356	51.3 (14.6)	344	51.5 (13.1)	-0.2 (-2.2 to 1.9)	0.880
3 month	256	72.2 (20.8)	274	66.3 (21.5)	5.8 (2.3 to 9.3)	0.001	317	77.6 (19.3)	313	71.8 (20.9)	5.5 (2.4 to 8.7)	<.001
12 month	238	78.8 (18.6)	255	70.9 (20.6)	7.6 (4.3 to 10.9)	<.001	290	85.3 (17.9)	280	78.5 (18.5)	6.4 (3.3 to 9.4)	<.001
24 month	217	79.6 (18.3)	216	73.0 (21.0)	6.4 (3.0 to 9.9)	<.001	272	84.9 (17.0)	258	78.9 (19.7)	5.4 (2.2 to 8.5)	<.001
36 month	162	77.7 (19.1)	152	75.9 (19.4)	2.8 (-0.9 to 6.5)	0.137	207	84.9 (15.3)	197	80.3 (18.5)	4.6 (1.3 to 7.9)	0.007
48 month	125	78.9 (18.1)	121	76.1 (19.5)	3.2 (-0.9 to 7.4)	0.127	160	85.1 (16.7)	149	79.4 (19.4)	5.4 (1.7 to 9.1)	0.005
60 month	88	76.3 (18.7)	90	75.6 (21.1)	1.7 (-3.3 to 6.6)	0.507	96	85.7 (15.0)	106	80.5 (20.0)	3.9 (-0.6 to 8.5)	0.092
All follow-up	1086	77.1 (19.3)	1108	71.8 (20.9)	4.6 (1.9 to 7.2)	<.001	1342	83.4 (17.6)	1303	77.5 (19.8)	5.2 (2.8 to 7.6)	<.001

AFEQT Summary Score, Baseline AFEQT Summary Score ≥70+												
	Women						Men					
	Catheter Ablation (N=112)		Drug Therapy (N=94)				Catheter Ablation (N=320)		Drug Therapy (N=334)			
	No.	Mean (SD)	No.	Mean (SD)	Mean adjusted difference (CA minus drug) (95% CI)	p-value	No.	Mean (SD)	No.	Mean (SD)	Mean adjusted difference (CA minus drug) (95% CI)	p-value
Baseline	112	81.1 (8.0)	94	81.1 (7.4)	0.0 (-2.3 to 2.3)	0.978	320	83.5 (8.3)	334	84.6 (8.8)	-1.1 (-2.4 to 0.2)	0.089
3 month	99	83.0 (14.4)	85	85.0 (13.4)	-1.9 (-5.7 to 1.8)	0.314	289	87.6 (13.3)	303	88.2 (12.5)	-0.8 (-2.9 to 1.3)	0.433
12 month	98	88.2 (14.3)	79	89.2 (11.5)	-0.7 (-4.0 to 2.6)	0.678	280	93.5 (9.0)	284	89.8 (11.6)	3.7 (1.8 to 5.5)	<.001
24 month	84	89.2 (12.2)	68	89.1 (14.2)	0.1 (-3.6 to 3.9)	0.947	274	93.0 (11.5)	251	90.1 (12.2)	2.6 (0.6 to 4.6)	0.012
36 month	62	90.9 (11.4)	55	89.8 (12.1)	0.8 (-2.9 to 4.5)	0.683	209	92.6 (10.3)	199	92.1 (10.2)	0.5 (-1.5 to 2.5)	0.646
48 month	43	89.9 (12.5)	38	88.4 (12.3)	1.1 (-3.5 to 5.6)	0.651	146	92.8 (9.8)	165	91.4 (11.2)	0.9 (-1.5 to 3.3)	0.465
60 month	35	91.3 (11.5)	24	89.5 (12.4)	3.2 (-2.3 to 8.6)	0.253	110	92.8 (12.0)	100	91.6 (10.9)	1.6 (-1.2 to 4.5)	0.260
All follow-up	421	88.0 (13.4)	349	88.2 (12.8)	0.4 (-2.4 to 3.2)	0.773	1308	91.8 (11.3)	1302	90.2 (11.7)	1.4 (-0.1 to 2.9)	0.074

AFEQT = Atrial Fibrillation Effect on Quality-of-life Questionnaire

Table S6. AFEQT Subdomains by Treatment Assignment and Sex

	Women						Men					
	Catheter Ablation (N=413)		Drug Therapy (N=406)				Catheter Ablation (N=695)		Drug Therapy (N=690)			
	No.	Mean (SD)	No.	Mean (SD)	Mean adjusted difference (CA minus drug) (95% CI)	p-value	No.	Mean (SD)	No.	Mean (SD)	Mean adjusted difference (CA minus drug) (95% CI)	p-value
**AFEQT Symptoms**												
Baseline	403	66.0 (21.7)	399	64.6 (24.1)	1.5 (-1.6 to 4.5)	0.341	669	75.1 (21.4)	671	76.1 (21.1)	-1.0 (-3.4 to 1.3)	0.396
3 month	357	84.1 (17.9)	357	78.7 (21.3)	5.4 (2.8 to 8.0)	<.001	609	87.9 (16.6)	622	85.6 (17.5)	2.2 (0.2 to 4.2)	0.028
12 month	336	88.4 (16.7)	330	81.9 (19.0)	6.1 (3.7 to 8.5)	<.001	571	93.3 (13.3)	570	88.5 (16.1)	4.8 (2.9 to 6.6)	<.001
24 month	300	88.5 (17.3)	280	84.4 (19.3)	3.6 (1.1 to 6.1)	0.006	539	93.7 (13.9)	494	90.8 (15.2)	2.5 (0.6 to 4.4)	0.011
36 month	214	89.7 (15.9)	198	85.6 (17.9)	4.0 (1.3 to 6.8)	0.004	408	93.7 (12.8)	385	91.1 (16.0)	2.6 (0.6 to 4.6)	0.013
48 month	164	87.4 (18.9)	155	86.2 (18.0)	1.4 (-1.9 to 4.6)	0.414	313	93.8 (13.1)	311	91.7 (15.6)	1.8 (-0.5 to 4.2)	0.128
60 month	121	87.1 (20.1)	112	84.7 (20.5)	1.9 (-1.9 to 5.7)	0.322	206	94.9 (12.4)	207	92.3 (14.4)	1.9 (-1.0 to 4.7)	0.195
All follow-up	1492	87.4 (17.6)	1432	82.8 (19.7)	3.7(1.9 to 5.6)	<.001	2646	92.4 (14.3)	2589	89.3 (16.3)	2.6 (1.2 to 4.0)	<.001
**AFEQT Daily Activities**												
Baseline	408	48.9 (26.1)	398	46.4 (24.5)	2.6 (-1.0 to 6.2)	0.162	675	60.9 (26.2)	678	61.1 (27.0)	-0.2 (-3.0 to 2.5)	0.865
3 month	353	70.3 (25.8)	359	64.1 (27.8)	6.0 (2.3 to 9.6)	0.001	608	79.3 (23.3)	615	75.8 (25.4)	3.3 (0.5 to 6.0)	0.022
12 month	337	76.4 (24.4)	329	68.7 (26.9)	7.2 (3.8 to 10.6)	<.001	569	86.6 (20.1)	565	80.5 (22.3)	5.6 (3.0 to 8.3)	<.001
24 month	294	76.3 (23.5)	283	69.2 (28.2)	6.6 (2.9 to 10.2)	<.001	548	85.6 (20.8)	508	79.8 (23.8)	4.9 (2.2 to 7.7)	<.001
36 month	222	75.3 (24.6)	205	72.4 (25.5)	3.4 (-0.6 to 7.3)	0.095	418	84.3 (21.1)	394	81.6 (22.1)	2.4 (-0.5 to 5.3)	0.111
48 month	170	74.8 (23.7)	159	72.1 (25.6)	3.4 (-1.0 to 7.9)	0.133	306	84.4 (20.4)	308	79.8 (23.5)	4.2 (0.9 to 7.5)	0.012
60 month	122	73.6 (25.1)	113	71.4 (26.5)	2.4 (-2.8 to 7.6)	0.364	204	85.1 (19.7)	204	81.3 (23.6)	2.2 (-1.7 to 6.2)	0.271
All follow-up	1498	74.4 (24.6)	1448	68.8 (27.1)	4.8 (2.0 to 7.7)	<.001	2653	84.0 (21.3)	2594	79.4 (23.6)	3.8 (1.6 to 5.9)	<.001
**AFEQT Treatment Concern**												
Baseline	406	60.8 (23.6)	399	60.0 (23.6)	0.8 (-2.3 to 4.0)	0.594	675	68.5 (22.4)	677	71.3 (21.5)	-2.8 (-5.2 to -0.4)	0.021
3 month	355	75.9 (20.8)	360	73.8 (22.0)	2.1 (-0.8 to 5.0)	0.159	610	82.6 (18.3)	617	81.3 (20.1)	0.8 (-1.4 to 3.0)	0.494
12 month	337	83.4 (18.5)	332	79.3 (21.4)	4.3 (1.6 to 6.9)	0.001	573	90.4 (14.7)	563	86.0 (17.2)	4.3 (2.3 to 6.4)	<.001
24 month	299	86.4 (16.6)	281	82.0 (20.6)	4.2 (1.5 to 6.9)	0.002	553	90.2 (14.3)	511	86.7 (17.4)	3.0 (1.0 to 5.0)	0.003
36 month	224	84.5 (18.3)	203	84.1 (19.0)	2.0 (-0.9 to 4.8)	0.178	417	91.0 (13.1)	397	88.8 (15.7)	2.0 (-0.1 to 4.1)	0.059
48 month	167	87.0 (16.8)	158	83.8 (19.0)	3.8 (0.7 to 7.0)	0.017	304	91.1 (14.3)	310	89.3 (15.7)	1.5 (-0.8 to 3.8)	0.202
60 month	120	86.4 (15.9)	112	83.2 (20.8)	4.1 (0.2 to 8.0)	0.038	205	91.8 (14.5)	202	88.2 (18.6)	2.7 (-0.3 to 5.6)	0.074

	Women						Men					
	Catheter Ablation (N=413)		Drug Therapy (N=406)				Catheter Ablation (N=695)		Drug Therapy (N=690)			
	No.	Mean (SD)	No.	Mean (SD)	Mean adjusted difference (CA minus drug) (95% CI)	p-value	No.	Mean (SD)	No.	Mean (SD)	Mean adjusted difference (CA minus drug) (95% CI)	p-value
All follow-up	1502	83.0 (18.8)	1446	79.9 (21.1)	3.4 (1.3 to 5.5)	0.001	2662	88.9 (15.6)	2600	86.0 (17.9)	2.4 (0.8 to 3.9)	0.003

AFEQT= Atrial Fibrillation Effect on Quality-of-life Questionnaire

Figure S1: AFEQT Symptoms Domain. AFEQT = Atrial Fibrillation Effect on Quality-of-life Questionnaire

Interval	Sex	Ablation	Drug	Adjusted Mean Difference
	Women<br>Men	(N=413)<br>(N=695)	(N=406)<br>(N=690)	
Baseline	Women	403	399	1.5 (-1.6 to 4.5)
	Men	669	671	-1.0 (-3.4 to 1.3)
3 Month	Women	357	357	5.4 (2.8 to 8.0)
	Men	609	622	2.2 (0.2 to 4.2)
12 Month	Women	336	330	6.1 (3.7 to 8.5)
	Men	571	570	4.8 (2.9 to 6.6)
24 Month	Women	300	280	3.6 (1.1 to 6.1)
	Men	539	494	2.5 (0.6 to 4.4)
36 Month	Women	214	198	4.0 (1.3 to 6.8)
	Men	408	385	2.6 (0.6 to 4.6)
48 Month	Women	164	155	1.4 (-1.9 to 4.6)
	Men	313	311	1.8 (-0.5 to 4.2)
60 Month	Women	121	112	1.9 (-1.9 to 5.7)
	Men	206	207	1.9 (-1.0 to 4.7)
All Follow-up	Women	1492	1432	3.7 (1.9 to 5.6)
	Men	2646	2589	2.6 (1.2 to 4.0)

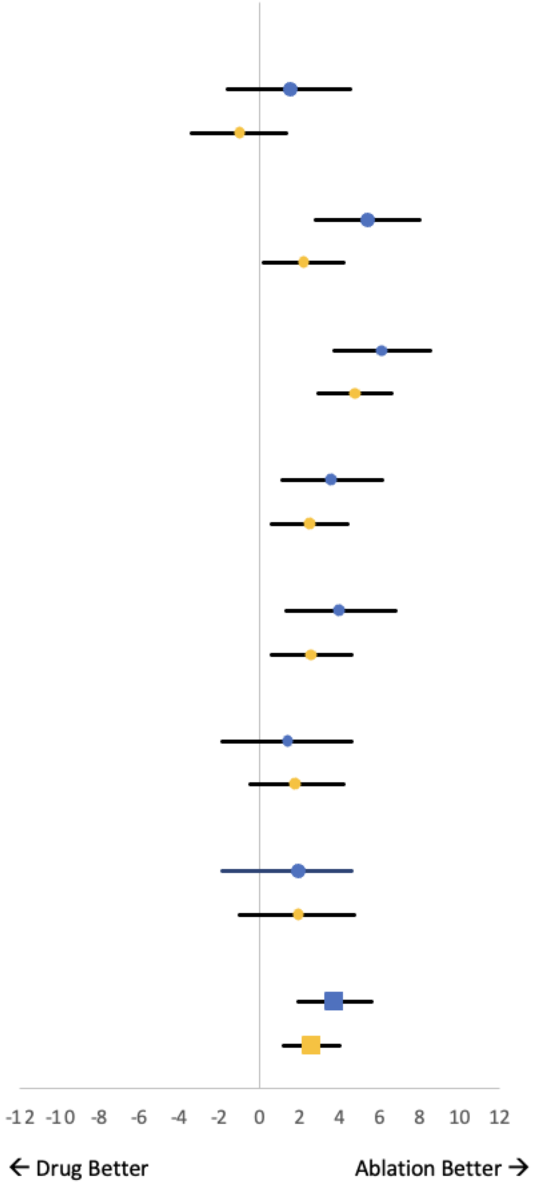

Figure S2: AFEQT Daily Activities Domain. AFEQT = Atrial Fibrillation Effect on Quality-of-life Questionnaire

Interval	Sex	Ablation	Drug	Adjusted Mean Difference
	Women<br>Men	(N=413)<br>(N=695)	(N=406)<br>(N=690)	
Baseline	Women	408	398	2.6 (-1.0 to 6.2)
	Men	675	678	-0.2 (-3.0 to 2.5)
3 Month	Women	353	359	6.0 (2.3 to 9.6)
	Men	608	615	3.3 (0.5 to 6.0)
12 Month	Women	337	329	7.2 (3.8 to 10.6)
	Men	569	565	5.6 (3.0 to 8.3)
24 Month	Women	294	283	6.6 (2.9 to 10.2)
	Men	548	508	4.9 (2.2 to 7.7)
36 Month	Women	222	205	3.4 (-0.6 to 7.3)
	Men	418	394	2.4 (-0.5 to 5.3)
48 Month	Women	170	159	3.4 (-1.0 to 7.9)
	Men	306	308	4.2 (0.9 to 7.5)
60 Month	Women	122	113	2.4 (-2.8 to 7.6)
	Men	204	204	2.2 (-1.7 to 6.2)
All Follow-up	Women	1498	1448	4.8 (2.0 to 7.7)
	Men	2653	2594	3.8 (1.6 to 5.9)

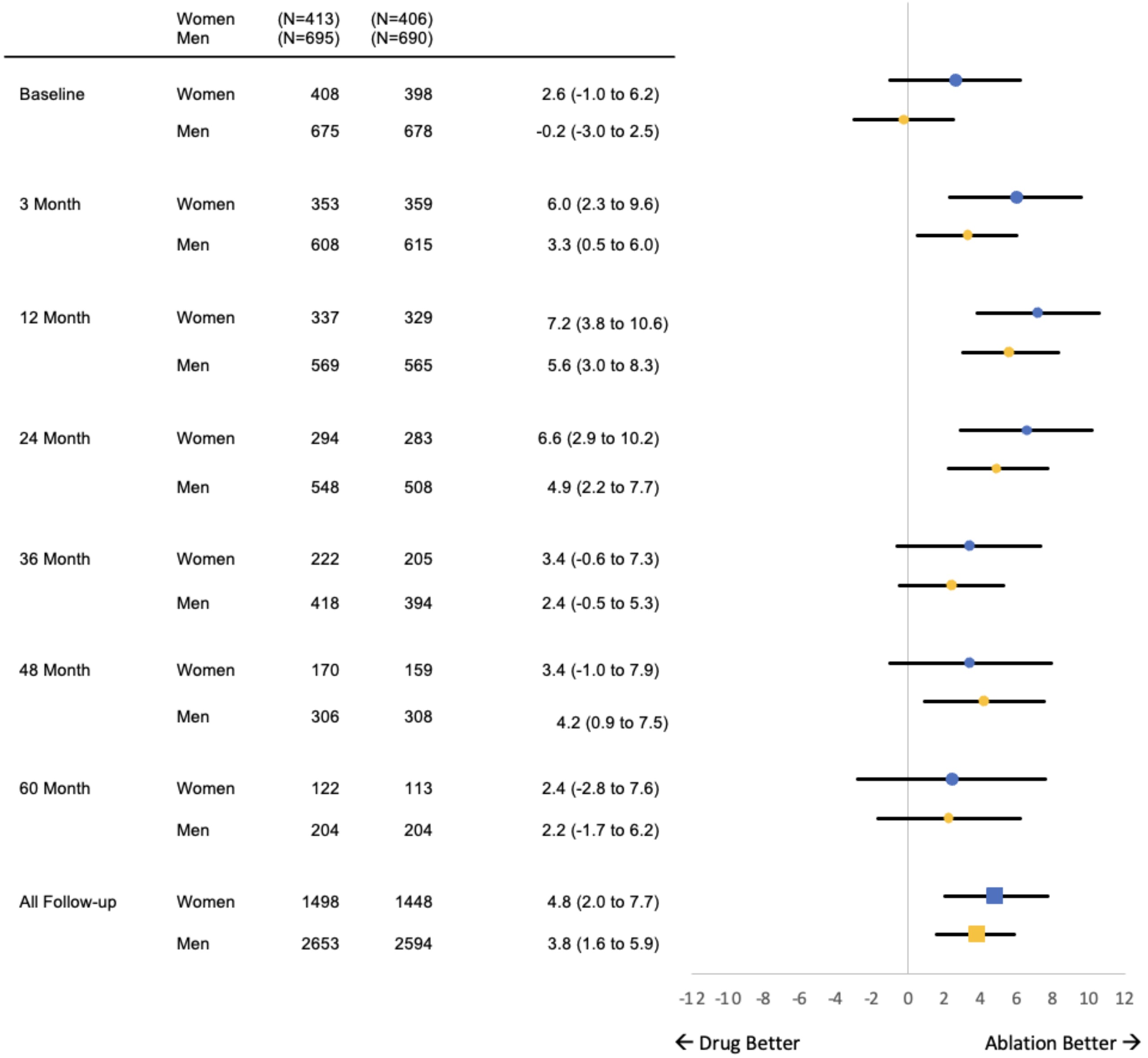

Figure S3: AFEQT Treatment Concern Domain. AFEQT = Atrial Fibrillation Effect on Quality-of-life Questionnaire

Interval	Sex	Ablation	Drug	Adjusted Mean Difference
	Women<br>Men	(N=413)<br>(N=695)	(N=406)<br>(N=690)	
Baseline	Women	406	399	0.8 (-2.3 to 4.0)
	Men	675	677	-2.8 (-5.2 to -0.4)
3 Month	Women	355	360	2.1 (-0.8 to 5.0)
	Men	610	617	0.8 (-1.4 to 3.0)
12 Month	Women	337	332	4.3 (1.6 to 6.9)
	Men	573	563	4.3 (2.3 to 6.4)
24 Month	Women	299	281	4.2 (1.5 to 6.9)
	Men	553	511	3.0 (1.0 to 5.0)
36 Month	Women	224	203	2.0 (-0.9 to 4.8)
	Men	417	397	2.0 (-0.1 to 4.1)
48 Month	Women	167	158	3.8 (0.7 to 7.0)
	Men	304	310	1.5 (-0.8 to 3.8)
60 Month	Women	120	112	4.1 (0.2 to 8.0)
	Men	205	202	2.7 (-0.3 to 5.6)
All Follow-up	Women	1502	1446	3.4 (1.3 to 5.5)
	Men	2662	2600	2.4 (0.8 to 3.9)

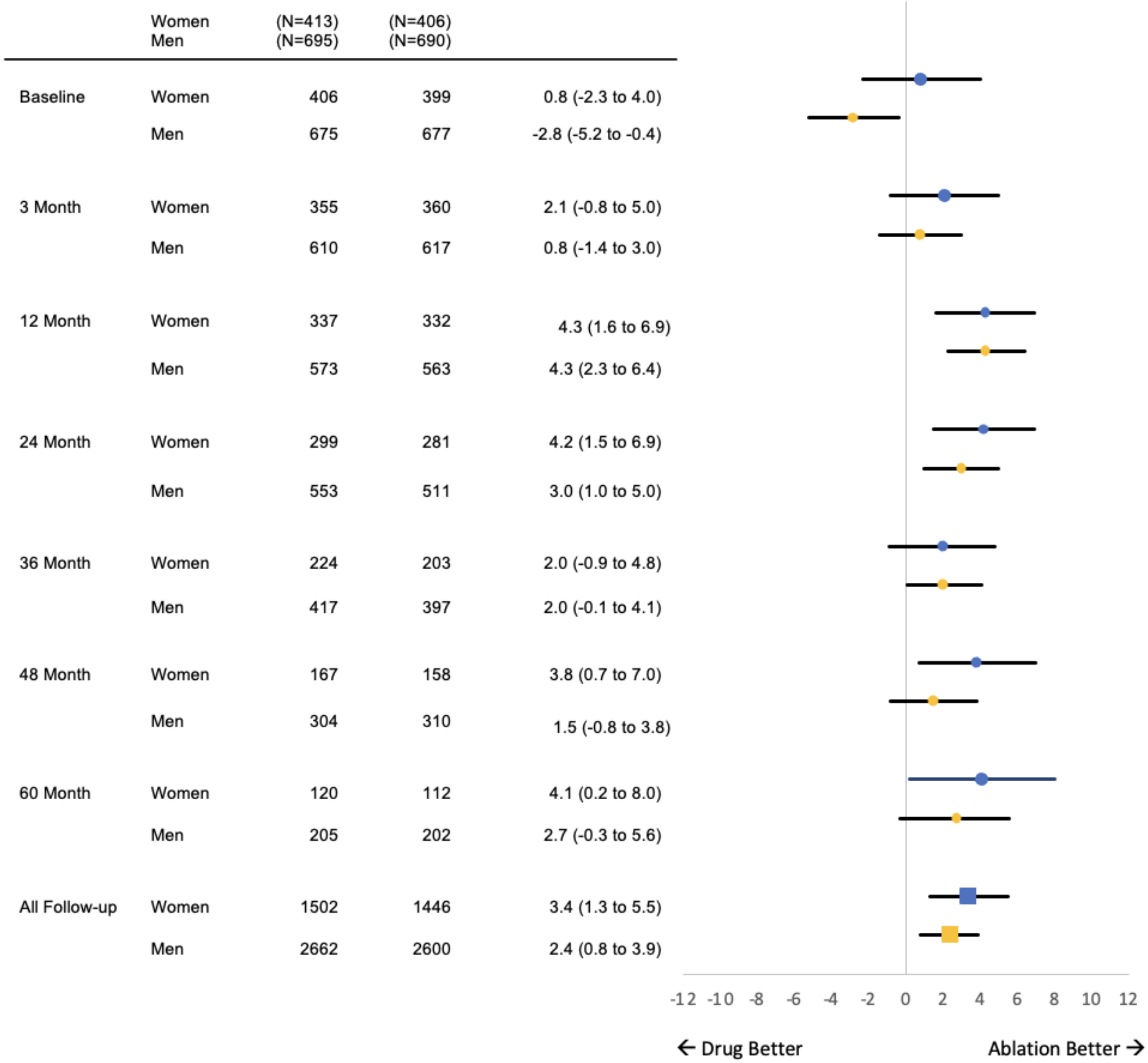

Figure S4: AFEQT Summary Score by Baseline AFEQT Summary Score <70. AFEQT = Atrial Fibrillation Effect on Quality-of-life Questionnaire

Interval	Sex	Ablation	Drug	Adjusted Mean Difference
	Women<br>Men	(N=296)<br>(N=356)	(N=306)<br>(N=344)	
Baseline	Women	296	306	0.4 (-1.8 to 2.7)
	Men	356	344	-0.2 (-2.2 to 1.9)
3 Month	Women	256	274	5.8 (2.3 to 9.3)
	Men	317	313	5.5 (2.4 to 8.7)
12 Month	Women	238	255	7.6 (4.3 to 10.9)
	Men	290	280	6.4 (3.3 to 9.4)
24 Month	Women	217	216	6.4 (3.0 to 9.9)
	Men	272	258	5.4 (2.2 to 6.5)
36 Month	Women	162	152	2.8 (-0.9 to 6.5)
	Men	207	197	4.6 (1.3 to 7.9)
48 Month	Women	125	121	3.2 (-0.9 to 7.4)
	Men	160	149	5.4 (1.7 to 9.1)
60 Month	Women	88	90	1.7 (-3.3 to 6.6)
	Men	96	106	3.9 (-0.6 to 8.5)
All Follow-up	Women	1086	1108	4.6 (1.9 to 7.2)
	Men	1342	1303	5.2 (2.8 to 7.6)

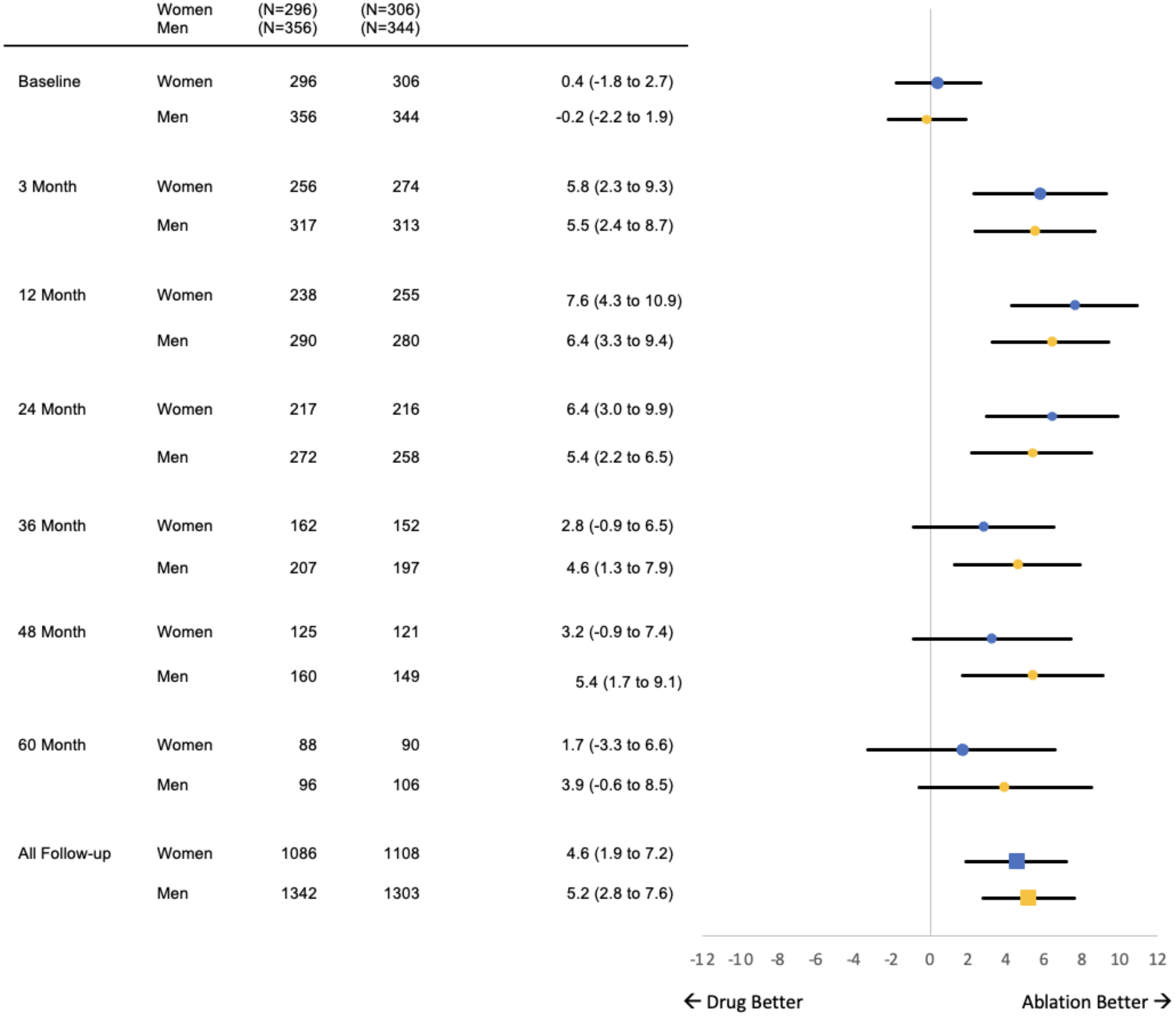

Figure S5: AFEQT Summary Score by Baseline AFEQT Summary Score ≥70. AFEQT = Atrial Fibrillation Effect on Quality-of-life Questionnaire

Interval	Sex	Ablation	Drug	Adjusted Mean Difference
	Women<br>Men	(N=112)<br>(N=320)	(N=94)<br>(N=334)	
Baseline	Women	112	94	0.0 (-2.3 to 2.3)
	Men	320	334	-1.1 (-2.4 to 0.2)
3 Month	Women	99	85	-1.9 (-5.7 to 1.8)
	Men	289	303	-0.8 (-2.9 to 1.3)
12 Month	Women	98	79	-0.7 (-4.0 to 2.6)
	Men	280	284	3.7 (1.8 to 5.5)
24 Month	Women	84	68	0.1 (-3.6 to 3.9)
	Men	274	251	2.6 (0.6 to 4.6)
36 Month	Women	62	55	0.8 (-2.9 to 4.5)
	Men	209	199	0.5 (-1.5 to 2.5)
48 Month	Women	43	38	1.1 (-3.5 to 5.6)
	Men	146	165	0.9 (-1.5 to 3.3)
60 Month	Women	35	24	3.2 (-2.3 to 8.6)
	Men	110	100	1.6 (-1.2 to 4.5)
All Follow-up	Women	421	349	0.4 (-2.4 to 3.2)
	Men	1308	1302	1.4 (-0.1 to 2.9)

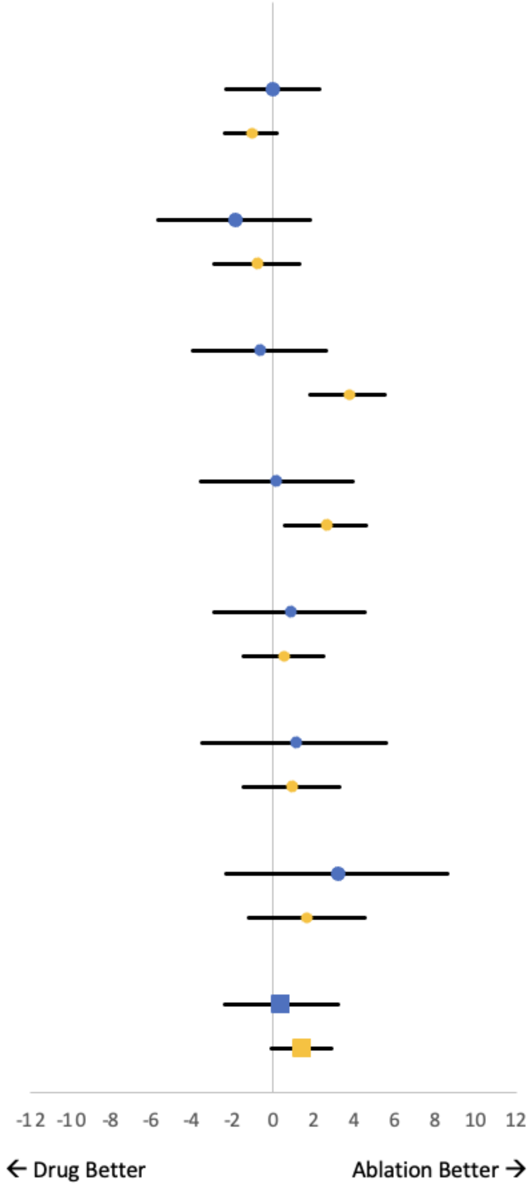

Supplement: Supplementary file 1 — Data S1 Tables S1–S6 Figures S1–S5 [file JAH3-12-e027871-s001.pdf]
